# Identification of strong candidate genes for backfat and intramuscular fatty acid composition in three crosses based on the Iberian pig

**DOI:** 10.1038/s41598-020-70894-2

**Published:** 2020-08-18

**Authors:** Daniel Crespo-Piazuelo, Lourdes Criado-Mesas, Manuel Revilla, Anna Castelló, José L. Noguera, Ana I. Fernández, Maria Ballester, Josep M. Folch

**Affiliations:** 1 Plant and Animal Genomics, Centre for Research in Agricultural Genomics (CRAG), CSIC-IRTA-UAB-UB Consortium, 08193 Bellaterra, Spain; 2 Departament de Ciència Animal i dels Aliments, Facultat de Veterinària, Universitat Autònoma de Barcelona (UAB), 08193 Bellaterra, Spain; 3 Genètica i Millora Animal, Institut de Recerca i Tecnologia Agroalimentàries (IRTA), 25198 Lleida, Spain; 4 Departamento de Mejora Genética Animal, Instituto Nacional de Investigación y Tecnología Agraria y Alimentaria (INIA), 28040 Madrid, Spain; 5 Departament de Genètica i Millora Animal, Institut de Recerca i Tecnologia Agroalimentàries (IRTA), 08140 Caldes de Montbui, Spain

**Keywords:** Animal breeding, Genome-wide association studies, Genetic markers, Quantitative trait loci

## Abstract

Meat quality has an important genetic component and can be modified by the fatty acid (FA) composition and the amount of fat contained in adipose tissue and muscle. The present study aimed to find genomic regions associated with the FA composition in backfat and muscle (*longissimus dorsi*) in 439 pigs with three different genetic backgrounds but having the Iberian breed in common. Genome-wide association studies (GWAS) were performed between 38,424 single-nucleotide polymorphisms (SNPs) covering the pig genome and 60 phenotypic traits related to backfat and muscle FA composition. Nine significant associated regions were found in backfat on the *Sus scrofa* chromosomes (SSC): SSC1, SSC2, SSC4, SSC6, SSC8, SSC10, SSC12, and SSC16. For the intramuscular fat, six significant associated regions were identified on SSC4, SSC13, SSC14, and SSC17. A total of 52 candidate genes were proposed to explain the variation in backfat and muscle FA composition traits. GWAS were also reanalysed including SNPs on five candidate genes (*ELOVL6*, *ELOVL7*, *FADS2*, *FASN*, and *SCD*). Regions and molecular markers described in our study may be useful for meat quality selection of commercial pig breeds, although several polymorphisms were breed-specific, and further analysis would be needed to evaluate possible causal mutations.

## Introduction

Meat quality depends on the consumer’s perception, which is subjected to the socio-demographic backgrounds of the consumer^1^, and is based on factors such as the nutritional value and the organoleptic properties of meat^2^. These factors can be modified by the fatty acid (FA) composition and the amount of fat that is contained in adipose tissue and muscle^3^. In addition, the consumer is becoming more concerned about the healthfulness of meat^1^. Certain saturated FAs (SFAs) raise the cholesterol and low-density lipoprotein (LDL) levels in blood, which increase the risk of suffering a cardiovascular disease^4,5^; whereas monounsaturated FAs (MUFAs) reduce plasma total LDL-cholesterol without affecting high-density lipoprotein (HDL) levels, which have an anti-atherogenic effect^4,5^. Polyunsaturated FAs (PUFAs), especially long-chain omega-3 fatty acids, also reduce LDL-cholesterol levels and the risk of cardiovascular disease^6^, but high amount of PUFAs in meat increase its susceptibility to oxidation, producing meat with undesirable sensory properties^3^. In contrast, a high concentration of MUFAs improve meat flavour^7^.

The Iberian pig is a breed characterized by its great meat quality, due to its high intramuscular fat (IMF) content with an increased proportion of MUFAs (mostly oleic acid) and a reduced quantity of PUFAs^8,9^. Conversely, other commercial breeds such as Pietrain and Landrace produce lean meat with a lower proportion of fat, although Pietrain carcasses exhibit a high ratio of intermuscular to subcutaneous fat^10^. Duroc pigs also exhibit a high intermuscular fat weight and develop a higher proportion of intramuscular fat than the commercial breeds^10^, which contain more PUFAs than the Iberian breed^11^. Altogether, the FA composition of adipose tissue and muscle in pigs show moderate to high heritability values^7,9,12^, revealing the importance of the genetic component in the variability of FA composition traits. Furthermore, FA composition is an expensive trait to measure and often require the slaughter of the animals. Polymorphisms associated with these traits can be used as genetic markers to evaluate the breeding value of an animal and increase the rate of genetic gain^13^.

Genetic selection in pigs has been intensifying thanks to high-density genotyping platforms, such as the PorcineSNP60 BeadChip (*Illumina*^®^)^14^ or the Axiom™ Porcine Genotyping Array (Affymetrix, Inc.)^15^. These chips allow the genotyping of markers distributed along the pig genome to perform Genome-Wide Association Studies (GWAS) for production traits. Through the use of GWAS, significant Quantitative Trait Loci (QTLs) in the pig genome have been identified for the FA composition in adipose tissue and muscle in several populations of crossed and purebred pigs such as Duroc, Landrace, Large White, and Erhualian^16–26^. In these studies, strong candidate genes related with lipid metabolism have been found for the FA composition in adipose tissue and muscle: ELOVL fatty acid elongases 6 and 7 (*ELOVL6*^17,18,21,22,24,25^ and *ELOVL7*^16,19,21,22^), fatty acid synthase (*FASN*)^21–23^, and stearoyl-CoA desaturase (*SCD*)^19–26^.

In this context, our group generated different crosses among Iberian purebred pigs and commercial breeds (IBMAP population) to identify QTLs associated with the FA composition in adipose tissue and muscle. In previous works, pigs from an IBMAP experimental backcross ((Iberian × Landrace) × Landrace) were genotyped with the PorcineSNP60 BeadChip (*Illumina*^®^) array^14^ and GWAS were performed for FA composition traits in adipose tissue and muscle using the *Sscrofa10.2* assembly^16,18^. In addition, GWAS have also been used in the IBMAP population to find QTLs associated with the expression of genes involved in lipid metabolism in adipose tissue and muscle^27,28^.

The present study aimed to identify genomic regions associated with FA composition of backfat and muscle in pigs with three different genetic backgrounds but having the Iberian breed in common using GWAS analyses.

## Material and methods

The methods reported on the present study were developed in the doctoral thesis of Crespo-Piazuelo^29^.

### Animal material

All animals used in the present work belong to three distinct pig backcrosses: (Iberian × Landrace) × Landrace (BC1_LD, n = 158), (Iberian × Duroc) × Duroc (BC1_DU, n = 143), and (Iberian × Pietrain) × Pietrain (BC1_PI, n = 138). Pigs were raised in an intensive system and fed ad libitum with a cereal-based commercial diet until slaughtered at 187.4 ± 10.1 days of age on NOVA GENÈTICA S. A. experimental farm (Lleida, Spain). Detailed information of generation schemes, diet, growth, and housing conditions of the three backcrosses is described in Martínez-Montes et al.^30^.

Samples of adipose tissue (backfat), diaphragm and *longissimus dorsi* muscle were collected at the commercial abattoir, snap-frozen in liquid nitrogen and stored at − 80 °C. A gas chromatography of methyl esters protocol^31^ was used to measure the FA profile (15 backfat FAs and 17 IMF FAs) of backfat samples taken between the third and the fourth ribs and 200 g of *longissimus dorsi* muscle. Then, the percentage of each individual FA methyl ester was calculated out of the total amount of FAs quantified. Total percentages of SFA, MUFA, and PUFA were obtained through the sum of the individual FAs that are included on their same section in Table 1. Metabolic and FA ratios were calculated from the ratio between individual FA percentages as it is shown in Table 1.

**Table 1:** Descriptive statistics including mean and SD of intramuscular fat and backfat fatty acid (FA) composition and FA indices in the merged dataset of the three backcrosses (n = 439).

Group	Trait	Name	Backfat		Intramuscular fat	
			Mean	SD	Mean	SD
SFA	C14:0	Myristic acid	1.14	0.11	1.17	0.24
	C16:0	Palmitic acid	23.06	1.48	22.99	1.51
	C17:0	Margaric acid	0.37	0.10	0.27	0.10
	C18:0	Stearic acid	14.76	1.79	14.21	1.44
	C20:0	Arachidic acid	0.28	0.05	0.26	0.11
	SFA	Saturated fatty acids	39.60	2.86	38.90	2.44
MUFA	C16:1(n-7)	Palmitoleic acid	1.53	0.25	2.61	0.50
	C16:1(n-9)	7-Hexadecenoic acid	0.38	0.10	0.36	0.11
	C17:1	Heptadecenoic acid	0.25	0.06	0.23	0.10
	C18:1(n-7)	Vaccenic acid	1.00	0.67	3.91	0.33
	C18:1(n-9)	Oleic acid	39.39	2.67	37.08	5.78
	C20:1(n-9)	Gondoic acid	1.02	0.16	0.82	0.20
	MUFA	Monounsaturated fatty acids	43.57	2.39	44.78	6.25
PUFA	C18:2(n-6)	Linoleic acid	14.76	2.78	11.92	5.01
	C18:3(n-3)	α-Linolenic acid	0.77	0.13	0.50	0.23
	C20:2(n-6)	Eicosadienoic acid	–	–	0.43	0.28
	C20:3(n-3)	Eicosatrienoic acid	–	–	2.53	2.04
	C20:3(n-6)	Dihomo-γ-linolenic acid	0.76	0.12	0.51	0.14
	C20:4(n-6)	Arachidonic acid	0.16	0.05	0.22	0.13
	PUFA	Polyunsaturated fatty acids	15.69	2.93	15.88	7.26
Metabolic ratios	MUFA/SFA	Ratio of MUFA to SFA	1.11	0.11	1.15	0.16
	PUFA/SFA	Ratio of PUFA to SFA	0.40	0.10	0.42	0.22
	MUFA/PUFA	Ratio of MUFA to PUFA	2.88	0.56	3.47	1.60
FA ratios	C16:1(n-7)/C16:0		0.07	0.01	0.11	0.02
	C18:1(n-7)/C16:1(n-7)		0.67	0.45	1.55	0.32
	C18:1(n-9)/C18:0		2.71	0.40	2.63	0.44
	C18:2(n-6)/C18:3(n-3)		19.29	1.99	26.27	11.76
	C20:1(n-9)/C20:0		3.72	0.46	3.42	1.09
	C20:3(n-6)/C18:2(n-6)		0.05	0.01	0.03	0.01
	C20:4(n-6)/C18:2(n-6)		0.01	0.00	0.19	0.08
	C20:4(n-6)/C20:3(n-6)		0.20	0.06	5.62	2.16

In total, 60 traits were analysed in backfat and IMF: 32 traits for FA percentages and 28 traits for indices of FA metabolism, including FA ratios for the activities of desaturases and elongases (Table 1).

### DNA extraction and single-nucleotide polymorphism (SNP) genotyping

DNA extraction was carried out with the phenol–chloroform method^32^ from the diaphragm of the 439 pigs. DNA concentration and purity were measured with a Nanodrop^®^ Spectrophotometer (ND-1000).

Two distinct systems were used for genotyping. The PorcineSNP60 BeadChip (*Illumina*^®^) array^14^ was employed to genotype 64,232 SNPs in the BC1_LD and BC1_PI animals using the Infinium^®^ HD Assay Ultra protocol (*Illumina*^®^) and results were visualized through the GenomeStudio software (2011.1 version, *Illumina*^®^). The Axiom™ Porcine Genotyping Array (Affymetrix, Inc.)^15^ was used for genotyping 658,692 SNPs in the BC1_DU pigs and genotypes were obtained and filtered with the Axiom™ Analysis Suite 2.0. For the GWAS analysis, we only considered the 45,845 SNPs that were found in common between both platforms and mapped in the *Sscrofa11.1* assembly. The SNPs on chromosome Y were removed as well as those SNPs with a minor allele frequency (MAF) < 5% or/and with missing genotypes > 5% using the PLINK software^33^ (1.90b5 version). Finally, a total of 38,424 SNPs remained for further analysis.

Furthermore, 21 SNPs in positional candidate genes were genotyped in the 439 pigs using Taqman OpenArray™ genotyping plates custom-designed in a QuantStudio™ 12K flex Real-Time PCR System (ThermoFisher Scientific). Of these 21 SNPs, five were SNPs located on the fatty acid desaturase 2 (*FADS2*) gene, seven were *ELOVL6*-SNPs, one SNP was located on the *FASN* gene, three were *ELOVL7*-SNPs, and five were SNPs located on the *SCD* gene.

### Genome-wide association studies (GWAS)

For the 439 pigs of the three backcrosses, GWAS were carried out between the 38,424 filtered SNPs and the FA composition and metabolic indices in backfat and IMF, described in Table 1. Thus, *GEMMA* software^34^ (0.96 version) was used to perform an univariate linear mixed model following this formula:

$${\text{y}}_{{{\text{ijklm}}}} = {\text{ Sex}}_{{\text{i}}} + {\text{Batch}}_{{\text{j}}} + {\text{ Backcross}}_{{\text{k}}} +\upbeta {\text{c}}_{{\text{l}}} + {\text{ u}}_{{\text{l}}} +\uplambda _{{\text{l}}} {\text{a}}_{{\text{m}}} + {\text{e}}_{{{\text{ijklm}}}} ,$$

where y_ijklm_ indicates the vector of phenotypic observations in the lth individual; sex (2 categories), batch based on slaughter day (14 categories) and backcross (3 categories) are fixed effects; β is a covariate coefficient with c being carcass weight; u_l_ is the infinitesimal genetic effect considered as random and distributed as N(0, Kσ_u_), where K is the numerator of the kinship matrix; λ_l_ is a − 1, 0, + 1 indicator variable depending on the lth individual genotype for the mth SNP; am represents the additive effect associated with the mth SNP; and e_ijklm_ is the residual effect. The kinship matrix was calculated with the genotypic information of the individuals using the centred relatedness matrix option of *GEMMA*^34^.

GWAS for candidate genes were performed following the previous linear mixed model between the 38,424 filtered SNPs, with the addition of the SNPs genotyped for each candidate gene, and the phenotypic traits that were associated with the region where the candidate gene was located. Five candidate gene-SNPs of the *Sus scrofa* chromosome (SSC) 2 region were included in the GWAS for the percentages of C16:1(n-9), C18:1(n-9) and C18:2(n-6), and the MUFA, PUFA, MUFA/PUFA and PUFA/SFA ratios in backfat. Seven candidate gene-SNPs of the SSC8 region were included in the GWAS for the percentages of C14:0, C16:0, and C16:1(n-7) in backfat. One candidate gene-SNP of the SSC12 region was included in the GWAS for the percentage of C14:0 in backfat. Three candidate gene-SNPs of the SSC16 region were included in the GWAS for the C20:1(n-9)/C20:0 ratio in backfat. Five candidate gene-SNPs of the SSC14 region were included in the GWAS for the C18:1(n-9)/C18:0 and MUFA/SFA ratios in IMF.

GWAS were also performed individually for each backcross following the previously described model without the backcross effect.

The false discovery rate (FDR) method of multiple testing described by Benjamini and Hochberg^35^ was used to measure the statistical significance for association studies at genome-wide level. The cut-off threshold for considering a SNP as significant was set at FDR ≤ 0.1. Quantile–quantile (Q–Q) plots and genomic inflation factors (λ) were obtained for all the GWAS with significant SNPs and are available as Supplementary Information S1.

The web based tool PhenoGram^36^ was used to visualize the differences obtained in the GWAS results between the merged dataset and each individual backcross.

### Region analysis, gene annotation and consequence prediction

If the distance between two significant SNPs was less than 10 Mb, they were grouped inside the same QTL interval. Only QTL intervals with a minimum of two SNPs were considered for further analyses. Intervals for different traits were merged if they overlapped. In addition, QTLs were defined and annotated at 1 Mb on each side of the previously defined intervals.

The extraction of the genes contained in the QTLs was performed with the BioMart tool^37^ from the Ensembl project (www.ensembl.org; release 92) using the *Sscrofa11.1* reference assembly. Furthermore, functional predictions of the significant SNPs were performed with the Variant Effect Predictor tool^38^ from the Ensembl project (release 92).

### Ethics statement

All animal procedures were performed according to the Spanish Policy for Animal Protection RD1201/05, which meets the European Union Directive 86/609 about the protection of animals used in experimentation. The experimental protocol was approved by the Ethical Committee of IRTA (*Institut de Recerca i Tecnologia Agroalimentàries*).

## Results and discussion

### GWAS results

A GWAS was performed using a total of 38,424 SNPs and the 60 phenotypic traits related with backfat and IMF FA composition (percentages, indices, and ratios) in a total of 439 pigs from three different backgrounds (BC1_DU, BC1_LD, and BC1_PI). In backfat, 98 significant associated SNPs located in nine *Sus scrofa* chromosomal regions were significantly associated with 12 traits (FDR ≤ 0.1; Table 2). In IMF, 39 SNPs located in six genomic regions were significantly associated with six traits (FDR ≤ 0.1; Table 3). In addition, no QTLs for backfat and IMF were found in common, indicating that the regulatory mechanisms affecting the FA composition of each tissue may be different.

**Table 2:** Description of the regions associated with the fatty acid composition in backfat and the candidate genes contained within them.

Region	Chr^a^	Start (bp)^b^	End (bp)^b^	No.SNPs^c^	Top SNP	MAF^d^	*p*-value	FDR	Trait	Candidate gene^e^
BF1	1	145,957,212	147,979,672	2	rs80899816	0.172	1.33 × 10^−6^	2.56 × 10^−2^	C16:1(n-9)	*GALR1*
BF2	2	0	12,764,773	24	rs81306755	0.391	2.51 × 10^−9^	7.90 × 10^−5^	C16:1(n-9); C18:1(n-9); C18:2(n-6); MUFA; MUFA/PUFA*; PUFA; PUFA/SFA	*CPT1A*; *ESRRA*; *FADS1*; *FADS2*; *FADS3*; *IGF2*; *INS*; *OSBP*; *PLA2G16*; *PNPLA2*; *PRPF19*; *SIRT3*; *ssc-mir-192*
BF3	4	80,446,371	82,565,259	4	rs80848071	0.415	5.47 × 10^−6^	1.40 × 10^−2^	MUFA/PUFA	*PRRX1*; *SLC19A2*
BF4	6	15,339,713	27,053,724	3	rs81322046	0.203	3.56 × 10^−7^	1.37 × 10^−2^	C20:3(n-6)	*BBS2*; *COQ9*; *GOT2*; *SLC12A3*
BF5	8	108,399,818	116,409,757	14	rs81403349	0.211	1.47 × 10^−10^	5.66 × 10^−6^	C14:0; C16:0*; C16:1(n-7)	*ELOVL6*; *HADH*; *PLA2G12A*
BF6	10	29,495,950	31,756,975	3	rs81423282; rs81423288	0.256	1.43 × 10^−5^	6.03 × 10^−2^	C16:1(n-9)	*NTRK2*; *RMI1*
BF7	10	51,874,658	53,920,353	2	rs80979357	0.095	7.51 × 10^−6^	4.81 × 10^−2^	C20:3(n-6)	*DNAJC1*; *PIP4K2A*
BF8	12	0	1,910,198	12	rs81308244	0.237	1.93 × 10^−10^	7.41 × 10^−6^	C14:0	*ASPSCR1*; *FASN*; *METRNL*; *NOTUM*
BF9	16	29,669,240	48,628,216	34	rs81297480; rs81458871	0.127	2.66 × 10^−8^	5.12 × 10^−4^	C20:1(n-9)/C20:0	*ELOVL7*; *GZMA*; *PIK3R1*; *PLPP1*

^a^*Sus scrofa* chromosome.

^b^Position on the chromosome where the region begins (start) and finishes (end).

^c^Number of significant SNPs found inside the region.

^d^Minor allele frequency.

^e^List of suggested genes for explaining part of the phenotypic variance of the trait.

*Indicates the most significant trait. If there is more than one associated trait on the region, the *p*-value and FDR of the top SNP refer to this trait.

**Table 3:** Description of the regions associated with the fatty acid composition in intramuscular fat and the candidate genes contained within them.

Region	Chr^a^	Start (bp)^b^	End (bp)^b^	No. SNPs^c^	Top SNP	MAF^d^	*p*-value	FDR	Trait	Candidate gene^e^
LD1	4	19,019,566	21,057,452	2	rs80910044	0.177	2.42 × 10^−6^	8.92 × 10^−2^	C20:4(n-6)/C20:3(n-6)	*ENPP2*; *EXT1*; *NOV*
LD2	4	122,756,546	124,979,309	3	rs81347340; rs80915252	0.437	1.18 × 10^−5^	7.65 × 10^−2^	C20:3(n-3)	*ABCD3*; *GCLM*
LD3	13	175,539,436	181,652,057	3	rs81441592	0.066	1.81 × 10^−6^	6.96 × 10^−2^	C20:3(n-3)	*LIPI*; *NRIP1*; *ssc-let-7c*
LD4	14	109,946,218	114,621,937	21	rs335655209	0.115	1.04 × 10^−7^	7.51 × 10^−4^	C18:1(n-9)/C18:0*; MUFA/SFA	*ELOVL3*; *SCD*
LD5	14	140,151,934	141,755,446	6	rs318740977; rs80814938; rs80883500	0.083	3.58 × 10^−6^	3.27 × 10^−2^	C18:0	*BNIP3*; *CYP2E1*; *ECHS1*
LD6	17	30,061,857	32,867,849	4	rs324135473	0.148	2.65 × 10^−6^	9.16 × 10^−2^	C20:0	*ABHD12*; *ACSS1*; *PANK2*; *ssc-mir-103–2*

^a^*Sus scrofa* chromosome.

^b^Position on the chromosome where the region begins (start) and finishes (end).

^c^Number of significant SNPs found inside the region.

^d^Minor allele frequency.

^e^List of suggested genes for explaining part of the phenotypic variance of the trait.

*Indicates the most significant trait. If there is more than one associated trait on the region, the *p*-value and FDR of the top SNP refer to this trait.

In the following sections, the candidate genes mapped in the genomic regions associated with the phenotypic traits of backfat and IMF are discussed in detail. The list of candidate genes is summarized in Table 2 for backfat traits and in Table 3 for IMF traits. All the significantly associated SNPs, and their predicted consequences, for the FA composition in backfat and in IMF are listed on the Supplementary Tables S1 and S2, respectively.

### QTLs for fatty acid composition in backfat and candidate genes

#### BF1

The 145.96–147.98 Mb region of SSC1 was associated with the C16:1(n-9) content in backfat (Table 2). The rs80899816 SNP was the most significant (*p*-value = 1.33 × 10^−6^) of the two SNPs comprised in this region, but both were located inside an intergenic region. Only one candidate gene, galanin receptor 1 (*GALR1*), was found in this region. This gene is a member of the galanin receptor family, which bind the neuropeptide hormone galanin^39^. Galanin regulates a range of biological functions such as food intake, neurogenesis, memory, and gut secretion^40^. Remarkably, the galanin-mediated signalling cascade has been associated with an activation of adipogenesis in high-fat diet induced obese mice^40,41^. In zebrafish, *GALR1* was up-regulated if animals were fed with a high fat fodder or with linoleic acid, participating in the accumulation of lipid droplets in cells^42^.

#### BF2

On SSC2, the 0–12.76 Mb region was associated with the abundance of three FAs in backfat (Table 2), C16:1(n-9), C18:1(n-9), and C18:2(n-6), and four metabolic ratios, MUFA, PUFA, MUFA/PUFA, and PUFA/SFA. In this QTL, 24 SNPs were found significantly associated with these seven traits and 13 genes were identified as candidates to explain the variation of those traits. The most significant SNP was rs81306755 (*p*-value = 2.51 × 10^−9^) for the MUFA/PUFA ratio. This significant SNP was located inside an intron of a novel gene (*ENSSSCG00000014565*) that was orthologous of the IFITM (interferon-induced transmembrane) protein family. However, fatty acid desaturases 1–3 (*FADS1*, *FADS2*, *FADS3*) are the most promising candidate genes found in this region to explain the variations in MUFA and PUFA content in backfat, specially *FADS2*. The essential FAs C18:2(n-6) and C18:3(n-3) are desaturated by FADS2, which can also desaturate C16:0 and C18:1(n-9)^43,44^. Other candidate gene, the *ssc-mir-192* gene, codifies for the miR-192 microRNA which impairs adipocyte triglyceride storage and suppresses the production of another desaturase, SCD^45^. The downregulation of another candidate gene, pre-mRNA processing factor 19 (*PRPF19*), also represses the expression of *SCD* and lipid droplet biogenesis in adipocytes^46^. In addition, three candidate genes found in this region are related with the oxidation of different FAs, carnitine palmitoyltransferase 1A (*CPT1A*), estrogen related receptor alpha (*ESRRA*), and phospholipase A2 group XVI (*PLA2G16*). The *CPT1A* gene encodes for a protein that is involved in mitochondrial β-oxidation of long-chain FAs^47^. ESRRA is an important regulator of the acyl-CoA dehydrogenase medium chain (*ACADM*) gene^48^, whose enzyme catalyses the initial rate limiting step in β-oxidation step of C4–C16 FAs with an optimum at C6-C8^49^. The *PLA2G16* gene encodes a major regulator of lipolysis in adipose tissue and through the regulation of FA oxidation in adipocytes may change the FA profile^50^. Conversely, other candidate genes found in this region have lipogenic effects, oxysterol binding protein (*OSBP*), insulin (*INS*) and insulin like growth factor 2 (*IGF2*). The overexpression of *OSBP* increased hepatic lipogenesis through insulin signalling pathways^51^. Thus, INS promotes FA uptake into cells and stimulates the expression of FA synthetic proteins^52^. In pigs, *IGF2* is responsible of 10–20% of the phenotypic variation in backfat thickness^53^. The last two candidate genes of this region were patatin like phospholipase domain containing 2 (*PNPLA2*) and sirtuin 3 (*SIRT3*). The enzyme encoded by *PNPLA2* participates in the hydrolysis of stored triglycerides in adipose tissue^54^, whereas the overexpression of one isoform of the *SIRT3* gene altered the FA composition in mouse skeletal muscle mitochondria including the MUFA/SFA ratio^55^. One or more candidate genes of SSC2 may be implicated in the genetic determination of the seven traits related with the FA composition in backfat. Therefore, further analysis of fine mapping would be needed to better elucidate the associations in this region.

#### BF3

The 80.45–82.57 Mb region of SSC4 was associated with the MUFA/PUFA ratio in backfat (Table 2). Located in an intergenic region, rs80848071 was the most significant SNP (*p*-value = 5.47 × 10^−6^) of this QTL. Two candidate genes were found inside this region: paired related homeobox 1 (*PRRX1*) and solute carrier family 19 member 2 (*SLC19A2*). PRRX1 is a transcription factor that negatively regulates adipogenesis in adipose tissue suppressing peroxisome proliferator activated receptor gamma (*PPARG*)^56^. Therefore, *PPARG* suppression may increase MUFAs and decrease PUFAs in adipocytes^57^. The protein encoded by the other candidate gene, *SLC19A2*, is a thiamine transporter which has been associated with human type 2 diabetes mellitus^58^. In addition, free FAs in plasma were reduced in rats that were fed with a thiamine deficient diet^59^.

#### BF4

The 15.34–27.05 Mb region of SSC6 was associated with the C20:3(n-6) abundance in backfat (Table 2). The rs81322046 SNP was the most significant (*p*-value = 3.56 × 10^−7^) and was located on an intergenic region. Four candidate genes were found in this region: glutamic-oxaloacetic transaminase 2 (*GOT2*), coenzyme 9 (*COQ9*), Bardet–Biedl syndrome 2 (*BBS2*), and solute carrier family 12 member 3 (*SLC12A3*). GOT2 is secreted from adipose tissue and is found in mitochondrion and cell surface facilitating uptake of long-chain free FAs^60^. In addition, GOT2 negatively regulates adipocyte differentiation^61^. COQ9 is also found in mitochondrion and acts as a lipid-binding protein playing an essential role for cellular respiration^62^. Finally, *BBS2* belongs to a family of genes that are involved in obesity^63^, while mutations in *SLC12A3* affect human serum level of low-density lipoprotein cholesterol^64^.

#### BF5

**Figure 1:**
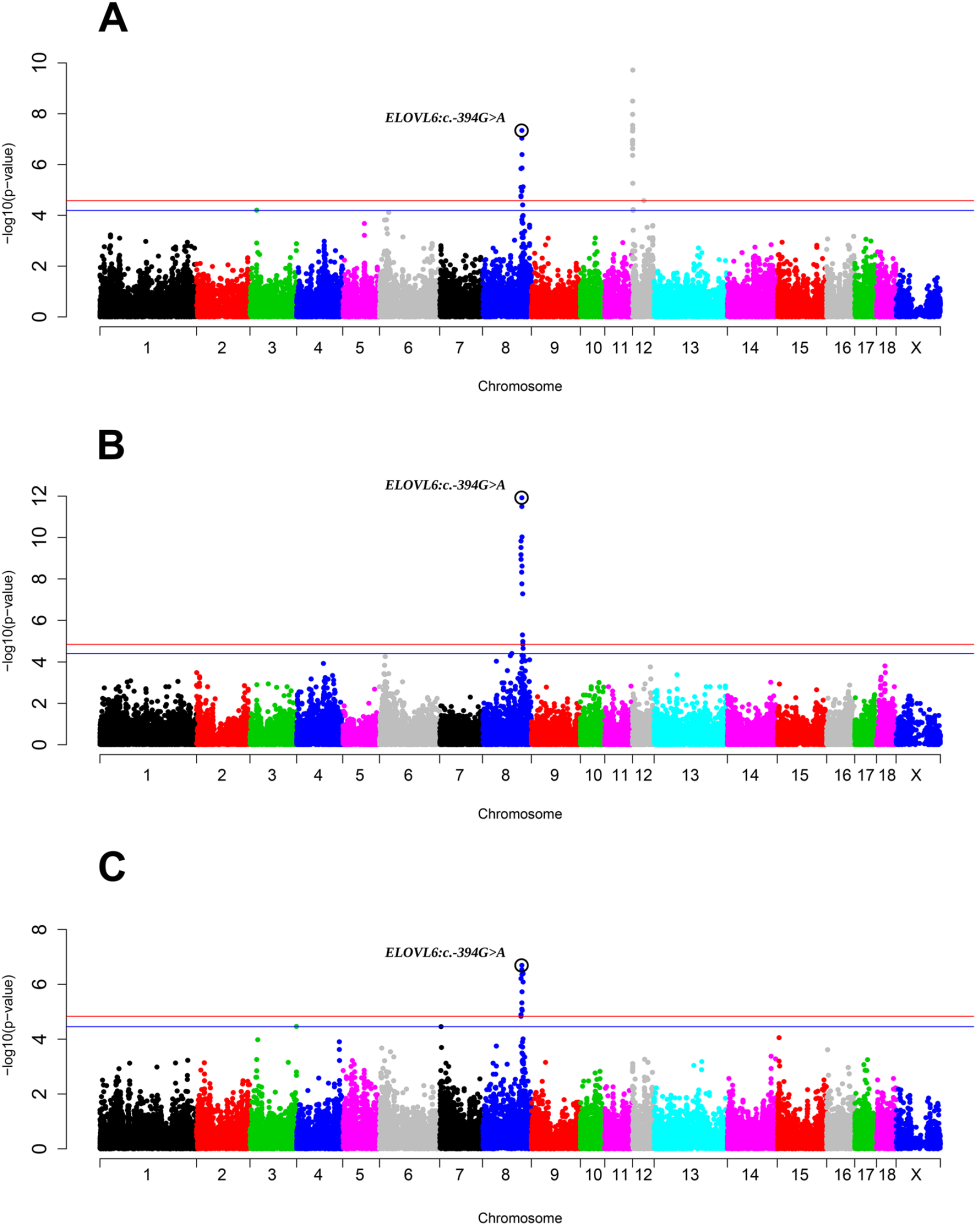
Manhattan plot representing the association analysis between the percentages of: C14:0 (**A**), C16:0 (**B**) and C16:1(n-7) (**C**) in backfat and SNPs distributed along the pig genome, including seven new genotyped polymorphisms for SSC8. The *ELOVL6:c.-394G*>*A* polymorphisms are included and labelled with a black circle. Red and blue lines indicate those SNPs that are below the genome-wide significance threshold (FDR ≤ 0.05 and FDR ≤ 0.1, respectively).

The 108.40–116.41 Mb region of SSC8 was associated with the FA content of C14:0, C16:0, and C16:1(n-7) in backfat (Fig. 1 and Table 2). Of the 14 significant SNPs found in this region, the most significant (*p*-value = 1.47 × 10^−10^) for the abundance of C16:0 in backfat was the rs81403349 variant, which was located on an intron of the ankyrin 2 (*ANK2*) gene. In this region, the *ELOVL6* gene was mapped, a promising candidate gene involved in the elongation of even C12-C16 SFAs and MUFAs^65^. In the BC1_LD animals, we have already reported a polymorphism in the promoter region of the *ELOVL6* gene (*ELOVL6:c.-533C*>*T), which was* strongly associated with the content of C16:0 and C16:1(n-7) in backfat and IMF^17^. Later on, the *ELOVL6:c.-394G*>*A* polymorphism was suggested as the causal mutation for the QTL on SSC8 that affects FA composition^66^. Other candidate genes found in this region were phospholipase A2 group XIIA (*PLA2G12A*) and hydroxyacyl-CoA dehydrogenase (*HADH*). PLA2G12A liberates C20:4(n-6) from phospholipids^67^, whereas HADH catalyses the oxidation of medium- and short-chain 3-hydroxy FAs^68^.

#### BF6

The 29.50–31.76 Mb region of SSC10 associated with the C16:1(n-9) abundance in backfat comprised three SNPs located in intergenic regions, being rs81423282 and rs81423288 (both *p*-values = 1.43 × 10^−5^) the most significant SNPs (Table 2). The two candidate genes found in this region, neurotrophic receptor tyrosine kinase 2 (*NTRK2*) and RecQ mediated genome instability 1 (*RMI1*), are involved in obesity^69,70^. However, further studies are required to discover how *NTRK2* or *RMI1* may be modifying the abundance in adipose tissue of a minor FA such as C16:1(n-9).

#### BF7

Another region (51.87–53.92 Mb) on SSC10 was associated with the abundance of C20:3(n-6) in backfat (Table 2). The most significant SNP of this QTL was rs80979357 (*p*-value = 7.51 × 10^−6^), which is located on an intron of the *DNAJC1* (DnaJ heat shock protein family (Hsp 40) member C1) gene. *DNAJC1* is involved in FA synthesis^71^ and has been found inside one genome-wide significant *locus* for subcutaneous adipose tissue in women^72^. On the other hand, another candidate gene located in this region was the *PIP4K2A* (phosphatidylinositol-5-phosphate 4-kinase type 2 alpha) gene. Liver of PIP4K2A-deficient mice were enriched in lipid droplets during fasting because autophagosomes failed to fuse with lysosomes at the rate needed^73^. Therefore, the impaired autophagy for recycling metabolites such as FAs may lead to cellular accumulation of C20:3(n-6) and other FAs in lipid droplets.

#### BF8

The 0–1.91 Mb region located at the beginning of SSC12 was associated with the C14:0 content in backfat (Table 2). Twelve SNPs were contained in this associated region and the most significant SNP was rs81308244 (*p*-value = 1.93 × 10^−10^), which was located in an intergenic region. Four candidate genes were found in this region, where *FASN* was the most promising. FASN produces predominantly C16:0 and, to a lesser extent, C14:0^74^. It is necessary to mention that along with *FASN*, the *ELOVL6* gene on SSC8 was also associated with the abundance of C14:0 in backfat (Fig. 1A), but *FASN* was not associated as *ELOVL6* with the percentages of C16:0 and C16:1(n-7) (Fig. 1B,C). In addition, *FASN* and *ELOVL6* showed a higher expression in the adipose tissue of BC1_LD pigs with low PUFA content^75^. Although *FASN* is the most promising candidate gene associated with the variation of C14:0 content, it is necessary to mention that C14:0 is mainly taken from the diet^76^ and it can be synthetized through other different pathways including C16:0 shortening^77^. In this sense, the proteins encoded by other candidate genes of this region may be affecting the C14:0 content in backfat. NOTUM (notum, palmitoleoyl-protein carboxylesterase) can bind to C14:1(n-5) and C16:1(n-7)^78^. In adipocytes, ASPSCR1 (ASPSCR1, UBX domain containing tether for SLC2A4) sequesters SLC2A4 (solute carrier family 2 member 4), also known as GLUT4, controlling glucose uptake^79^, while METRNL (meteorin like, glial cell differentiation regulator) promotes lipid metabolism and insulin sensitization^80^.

#### BF9

**Figure 2:**
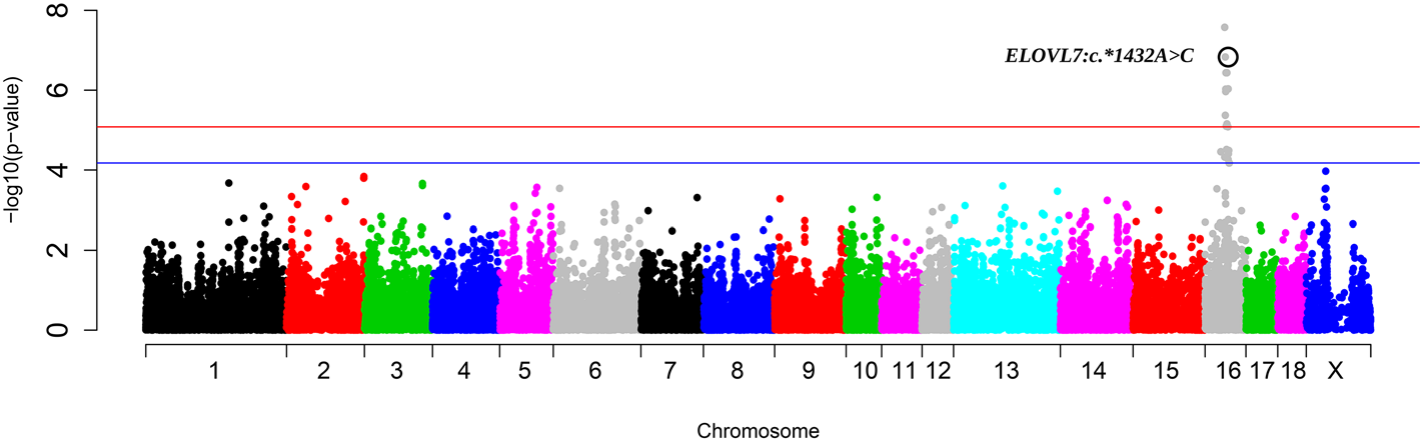
Manhattan plot representing the association analysis between the C20:1(n-9)/C20:0 ratio in backfat and SNPs distributed along the pig genome, including three new genotyped polymorphisms for SSC16. The ELOVL7:c.*1432A>*G* polymorphism is included and labelled with a black circle. Red and blue lines indicate those SNPs that are below the genome-wide significance threshold (FDR ≤ 0.05 and FDR ≤ 0.1, respectively).

The 29.67–48.63 Mb region of SSC16 was associated with the differences in the C20:1(n-9)/C20:0 ratio in backfat (Fig. 2 and Table 2). A total of 34 SNPs associated with the C20:1(n-9)/C20:0 ratio in backfat were found in this region. The two most significant SNPs, rs81297480 and rs81458871 (both *p*-values = 2.66 × 10^−8^), were intergenic variants. Four candidate genes were found in this region, *ELOVL7*, *GZMA* (granzyme A), *PIK3R1*, and *PLPP1* (phospholipid phosphatase 1). The *ELOVL7* gene is a strong candidate gene to explain the variation in C20:0 and C20:1(n-9) due to its protein function, which elongates C16-C20 FAs, with a preference toward C18 FAs^81^. A higher expression of *GZMA* was observed in the mesenteric adipose tissue of beef cattle with low gain when compared with high gain animals^82^. The *PIK3R1* gene regulates glucose import^83^ and in rat ovaries, the PIK3R1/AKT pathway has been involved in stearoyl-CoA desaturase 2 (*SCD2*) expression^84^. The protein encoded by *PLPP1* converts lipids such as phosphatidic acid and LPA to diacylglycerols^85^.

### QTLs for fatty acid composition in intramuscular fat and candidate genes

#### LD1

Two regions on SSC4 were associated with the FA composition of IMF. The first region (19.02–21.06 Mb) was associated with the C20:4(n-6)/C20:3(n-6) ratio (Table 3). The rs80910044 variant, located inside an intergenic region, was the most significant SNP (*p*-value = 2.42 × 10^−6^) of this QTL. Inside this region the ectonucleotide pyrophosphatase/phosphodiesterase 2 (*ENPP2*) gene was identified, also known as autotaxin (*ATX*). The protein encoded by *ENPP2* converts lysophosphatidylcholines to lysophosphatidic acids (LPAs) formed by different FAs such as C18:1(n-9) or C20:4(n-6)^86^. Furthermore, LPA is a signalling lipid involved in growth-factor like responses^87^ and also participates in FA esterification being a precursor of the triglycerides that are deposited in IMF^88^. Other candidate genes in the SSC4 region that could be modulating the C20:4(n-6)/C20:3(n-6) ratio were exostosin glycosyltransferase 1 (*EXT1*) and nephroblastoma overexpressed (*NOV*). The mutation of hepatic *EXT1* increased the levels of plasma triglycerides in mice^89^, whereas *NOV* was involved in the development of obesity^90^ and the suppression of myogenesis^91^.

#### LD2

The second region (122.76–124.98 Mb) on SSC4 was associated with the C20:3(n-3) content in IMF (Table 3). The two most significant SNPs (both *p*-values = 1.18 × 10^−5^) of this QTL were rs81347340 and rs80915252. While the rs81347340 variant was located in an intergenic region, the rs80915252 variant was a splice region variant and a synonymous variant of the coiled-coil domain containing 18 (*CCDC18*) gene. Two candidate genes were found in this region: ATP binding cassette subfamily D member 3 (*ABCD3*) and glutamate-cysteine ligase modifier subunit (*GCLM*). *ABCD3* encodes for a protein that imports free FAs into peroxisomes where substrates are chain-shortened by β-oxidation^92^. In particular, ABCD3 has been reported to preferentially transport hydrophilic unsaturated FAs such as C20:3(n-3)^92^. The other candidate gene, *GCLM*, plays a role in the metabolism of dietary lipids and mice with *GCLM*-deficiency were protected from weight gain and adipose deposition^93^.

#### LD3

Three candidate genes were located in the 175.54–181.65 Mb region of SSC13 associated with the abundance of C20:3(n-3) in IMF (Table 3). The most significant variant found inside this region was the rs81441592 intergenic variant (*p*-value = 1.81 × 10^−6^). The lipase I (*LIPI*) gene encodes for a phospholipase that breaks down phosphatidic acid into LPA. In this sense, LIPI may have a similar role as the aforementioned ENPP2 in LPA production which would affect the FAs that are deposited in IMF^88^. The nuclear receptor interacting protein 1 (*NRIP1*) gene is involved in fat accumulation^94^ and lipolysis^95^. The last candidate gene (*ssc-let-7c*) is transcribed into a microRNA, miR-let-7c, that regulates muscle growth in pigs, whereas other members of its family (miR-let-7a and miR-let-7e) regulate lipid deposition^96^.

#### LD4

The first region (109.95–114.62 Mb) found on SSC14 was associated with two metabolic ratios in IMF, C18:1(n-9)/C18:0 and MUFA/SFA (Table 3). This QTL comprised a total of 21 significant associated SNPs, being the rs335655209 variant the most significant SNP (*p*-value = 1.04 × 10^−7^) for the C18:1(n-9)/C18:0 ratio as well as one of the most significant SNPs (*p*-value = 1.11 × 10^−6^) for the MUFA/SFA ratio. The rs335655209 variant was located in an intron of the *BTRC* (beta-transducin repeat containing E3 ubiquitin protein ligase) gene. These two desaturation ratios may be modulated by the *SCD* gene found in this region. SCD participates in the biosynthesis of C18:1(n-9) by desaturating C18:0^97^. Polymorphisms in *SCD* have been related with differences in FA composition and desaturation ratios in swine backfat and IMF^98,99^. However, the *ELOVL3* (ELOVL fatty acid elongase 3) gene is also inside this region and may affect FA composition through the synthesis of C20-C24 SFAs and MUFAs^100^. Therefore, *SCD* and *ELOVL3* are strong candidate genes to modulate the FA composition in muscle.

#### LD5

The second region (140.15–141.76 Mb) found on SSC14 was associated with the C18:0 abundance in IMF (Table 3). This region was comprised of six significant SNPs and three of them (rs318740977, rs80814938 and rs80883500) were the most significant variants (*p*-value = 3.58 × 10^−6^) for this QTL. The rs318740977 and rs80814938 SNPs were intronic variants of the *KNDC1* (kinase non-catalytic C-lobe domain containing 1) gene, whereas the rs80883500 SNP was located inside an intron of the *CALY* (calcyon neuron specific vesicular protein) gene. Three candidate genes were found inside this region: enoyl-CoA hydratase, short chain 1 (*ECHS1*); cytochrome P450 family 2 subfamily E member 1 (*CYP2E1*), and BCL2 interacting protein 3 (*BNIP3*). ECHS1 is involved in mitochondrial FA β-oxidation, but its activity is linked to short-chain FAs^101^. In the same manner, CYP2E1 has preference for short SFAs and long unsaturated FAs, showing no C18:0 hydroxylase activity^102^. In addition, CYP2E1 activity was inhibited by PUFAs but no by C16:0 and C18:0^103^. Therefore, mutations in *ECHS1* or *CYP2E1* may increase the C18:0 abundance in IMF through the modification of short SFAs metabolism. On the other hand, *BNIP3* may be responsible of the differences in C18:0 as well. The *BNIP3* gene is a mitophagy regulator that, when silenced, suppressed *FASN*-mediated free FA synthesis^104^.

#### LD6

The 30.06–32.87 Mb region of SSC17 was associated with the C20:0 content in IMF (Table 3). The most significant SNP of this region was rs324135473 (*p*-value = 2.65 × 10^−6^), located in an intergenic region. The acyl-CoA synthase short chain family member 1 (*ACSS1*) gene is located within this region. *ACSS1* was differentially expressed in bulls with extreme FA composition in muscle^105^. Therefore, in our material, ACSS1 may be increasing the SFA amount through the transformation of acetyl-CoA into FAs. Located in the SSC17 region was another relevant gene, pantothenate kinase (*PANK2*). Humans with mutations in *PANK2* present lower levels of some FAs compared to controls^106^. Furthermore, inside an intron of *PANK2*, it is located the *ssc-mir-103-2* gene, which is transcribed into the miR-103-2 microRNA. In adipocytes, miR-103 accelerates adipogenesis and increases the expression of lipid metabolism related genes such as fatty acid binding protein 4 (*FABP4*) and adiponectin, C1Q and collagen domain containing (*ADIPOQ*)^107^. The last candidate gene found in this SSC17 region that may be modulating the C20:0 content was abhydrolase domain containing 12 (*ABHD12*). ABHD12 has monoacylglycerol lipase activity and preferentially hydrolyses 2-arachidonoylglycerol, which is an ester of C20:4(n-6) and glycerol^108^.

### Comparison between merged dataset GWAS and backcross-specific GWAS

**Figure 3:**
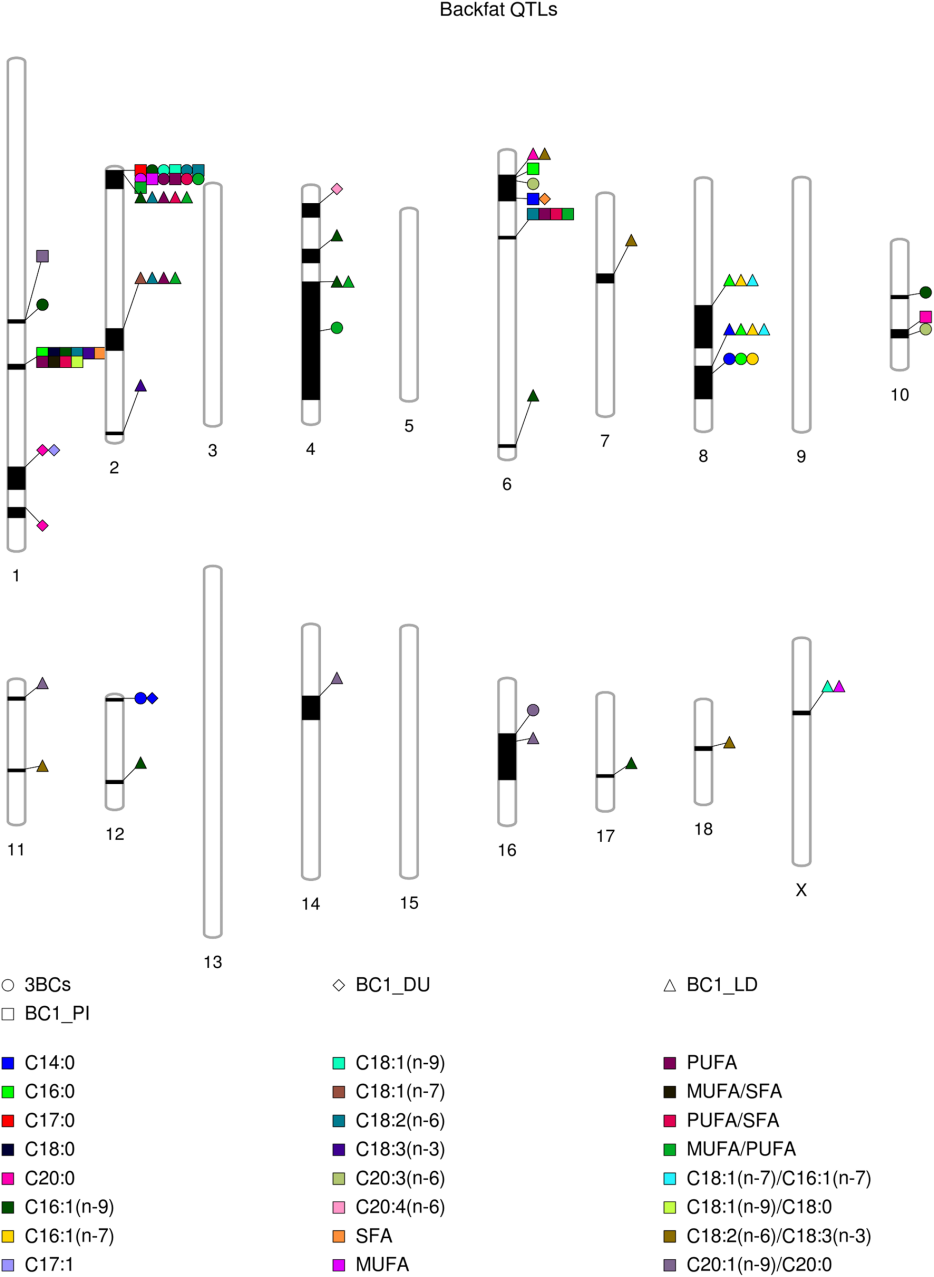
Comparison between the associated regions along pig chromosomes for backfat FA composition in the merged dataset and in each backcross individually. The shape indicates the backcross or the merged dataset and the colour indicates the phenotypic trait as it is indicated in the legend.

**Figure 4:**
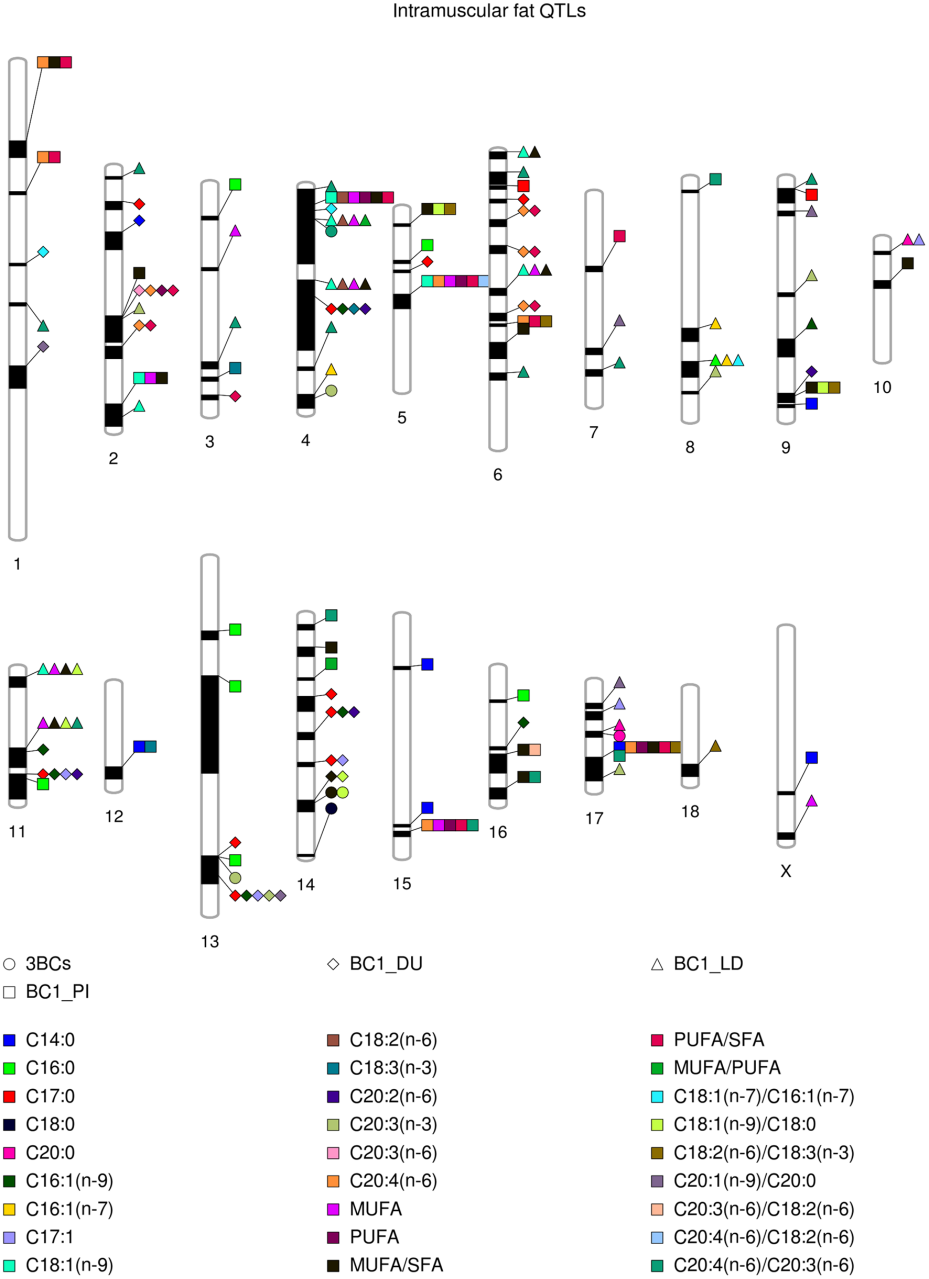
Comparison between the associated regions along pig chromosomes for intramuscular FA composition in the merged dataset and in each backcross individually. The shape indicates the backcross or the merged dataset and the colour indicates the phenotypic trait as it is indicated in the legend.

Backcross-specific GWAS for the FA composition in backfat found 18 associated regions in BC1_LD, seven in BC1_PI, and five in BC1_DU (Fig. 3 and Supplementary Table S3), whereas the backcross-specific regions found for the FA composition in IMF were 35 in BC1_PI, 33 in BC1_LD, and 24 in BC1_DU (Fig. 4 and Supplementary Table S3). Hence, more significant associated regions were found for the FA profile in IMF than in backfat. In backfat, all the regions for FA composition found in the merged dataset overlapped with at least one backcross-specific region. There was overlapping on the first region of SSC2 (0–10.77 Mb) between BC1_LD and BC1_PI for C18:2(n-6), PUFA and MUFA/PUFA in backfat. In addition, two SSC6 regions were shared between BC1_PI and BC1_LD (12.14–18.79 Mb) and between BC1_PI and BC1_DU (25.05–27.96 Mb) for different FA composition traits in backfat (C16:0, C20:0 and C18:2(n-6)/C18:3(n-3) on the first SSC6 region, and C14:0 and SFA on the second one). Similarly, all the intramuscular FA composition regions found in the merged dataset overlapped with at least one backcross-specific region, except for the last region of SSC14 (140.15–141.76 Mb). Two regions associated with the intramuscular FA composition were overlapping in the three backcrosses, although the number of associated traits varied among backcrosses: the 85.56–101.25 Mb region on SSC2 (MUFA/SFA on BC1_PI; C20:3(n-6), C20:4(n-6), PUFA, and PUFA/SFA on BC1_DU, and C20:3(n-3) on BC1_LD), and the 10.50–45.32 Mb region on SSC4 (C18:1(n-9), C18:2(n-6), MUFA, PUFA, MUFA/SFA and PUFA/SFA on BC1_PI; C18:1(n-7)/C16:1(n-7) on BC1_DU, and C18:1(n-9), C18:2(n-6), MUFA, and MUFA/PUFA on BC1_LD). Furthermore, a total of eight regions associated with the intramuscular FA composition were overlapping between two backcrosses, on SSC2 (136.67–149.91 Mb between BC1_LD and BC1_PI), on SSC4 (54.28–95.60 Mb between BC1_DU and BC1_LD), on SSC9 (5.39–14.20 Mb between BC1_LD and BC1_PI), a second region on SSC9 (124.17–130.17 Mb between BC1_DU and BC1_PI), on SSC11 (46.32–58.27 Mb between BC1_DU and BC1_LD), a second region on SSC11 (61.64–76.84 Mb between BC1_DU and BC1_PI), on SSC13 (174.35–191.27 Mb between BC1_DU and BC1_PI), and on SSC17 (44.22–58.14 Mb between BC1_LD and BC1_PI). In summary, using different genotypic backgrounds reduced the number of significant associated regions increasing the relevance and robustness of the detected ones. However, most of the associated regions found in the merged dataset were driven by one backcross and then, mixing backcrosses resulted in the loss of *loci* associated to a specific backcross.

### Comparison with other studies

Certain regions of the pig genome have been commonly found in association with FA composition traits in adipose tissue and muscle, where strong candidate genes are located. In accordance with previous studies, the region on SSC8 was associated with the abundance of C16:0, C16:1(n-7), and C18:1(n-9) in adipose tissue and muscle, reporting *ELOVL6* as candidate gene^21,22,24,25^. On SSC16, the *ELOVL7* gene was also proposed as a candidate gene to explain the GWAS signals associated with the C20:0 abundance and several metabolic indices, such as C20:1(n-9)/C20:0, in the abdominal fat and IMF of different pig populations^19,21,22^. A similar QTL signal was detected on SSC12, where the *FASN* gene was located, on GWAS performed for the abundance of C14:0 in adipose tissue and muscle in Erhualian and Duroc pigs^21,23^. The signal found on SSC14 for the percentages of C16:0, C16:1(n-7), C18:0, C18:1(n-9), SFAs, and MUFAs was also found on GWAS performed in different pig populations for FA composition in adipose tissue and IMF, which reported *SCD* and *ELOVL3* as candidate genes^19–26^. On a previous study in the BC1_LD animals, the *FADS* genes were proposed as potential candidate genes for the SSC2 QTL associated with the percentages of C16:1(n-9), C18:2(n-6), C18:3(n-3), and PUFAS in backfat^109^. In other studies, *FADS2* was also proposed as a strong candidate gene to explain the variation of two metabolic indices (C20:3(n-6)/C18:2(n-6) and C20:4(n-6)/C20:3(n-6)) in the IMF of Erhualian pigs^22^, and the C20:4 to C18:2 ratio in the muscle of Duroc pigs^110^.

In a previous study with the BC1_LD pigs^16^, oleic and linoleic acid content and PUFA and PUFA/SFA QTLs were found for the 145.96–147.98 Mb region on SSC1, although this region was only associated with 7-hexadecenoic acid content in the merged dataset of the three backcrosses. The 80.45–82.57 Mb region on SSC4 has been associated in several studies with oleic and linoleic acid content^111–113^. In the present study, this SSC4 region was associated with the MUFA/PUFA ratio, being the *PRRX1* gene a clear candidate of this region due to its role in adipogenesis^56^. In our study, two regions were associated with dihomo-γ-linolenic acid content, although the 15.34–27.05 Mb region on SSC6 was previously associated with palmitic and eicosadienoic acid content^19,114^, and the 51.874–53.92 Mb region on SSC10 was previously associated with linoleic acid content^114^.

The 19.02–21.06 region on SSC4 was associated with C20:4(n-6)/C20:3(n-6) content in muscle, despite it has been described QTLs for linoleic acid content in the BC1_LD pigs and other population^18,114^. On SSC4, there was found another region (122.76–124.98 Mb) associated with eicosatrienoic acid content. However, this region has also been associated with the content of different FAs (palmitic, margaric and oleic acid)^19,114,115^. A strong candidate gene of this region was the *ABCD3* gene, which acts as a transporter of FAs^92^. The 30.06–32.87 Mb region on SSC17 was previously reported in association with the margaric acid content^23^. Conversely, this same region was associated with the arachidic acid content in our study.

Regions 29.50–31.76 Mb on SSC10 in backfat, and 175.54–181.65 Mb on SSC13 and 140.15–141.76 Mb on SSC14 in muscle, were not found previously in association with any fat composition trait^116^.

### GWAS for candidate genes

As some of the QTL signals found in our study were previously reported in other GWAS for FA composition in adipose tissue and muscle in several swine populations^16–26^, we decided to genotype polymorphisms in the most recurrent candidate genes reported in these studies: *FADS2*, *FASN*, *ELOVL6*, *ELOVL7* and *SCD*. A total of 21 SNPs were genotyped among these positional candidate genes. For each QTL signal, GWAS were performed again adding the polymorphisms genotyped on each candidate gene to the 38,424 SNPs already used. The results of these analyses for these five candidate genes are discussed in detail in this section.

#### FADS2

GWAS for the abundance of three FAs in backfat, C16:1(n-9), C18:1(n-9), and C18:2(n-6), and four metabolic ratios, MUFA, PUFA, MUFA/PUFA, and PUFA/SFA were reanalysed including five SNPs of the *FADS2* gene. These SNPs were found in the genome sequences of the BC1_LD founders as described in Revilla et al.^117^. Two of the SNPs were located in the sixth exon (rs326796107 (*FADS2:c.789T*>*G*)) and the eighth exon (rs335011456 (*FADS2:c.900C*>*T*)) of the *FADS* gene, while the other three were upstream region variants (rs331050552, rs336076510 and rs344625804). These three upstream variants were also detected on a Duroc population^110^, where rs331050552 and rs336076510 were described to be fully linked with the rs321384923 variant, showing a strong association with the C18:2/C20:4 ratio in muscle. However, no association was found between the five *FADS2*-SNPs and the seven traits in the GWAS performed on the merged dataset of our population. Conversely, on the GWAS conducted in the BC1_LD backcross, *FADS2:c.789T*>*G* was significantly associated with the content of C18:2(n-6) (*p*-value = 5.81 × 10^−6^) and the PUFA (*p*-value = 7.30 × 10^−6^) and MUFA/PUFA (*p*-value = 1.50 × 10^−6^) ratios in backfat, whereas it was not the strongest association found on the SSC2 QTL of BC1_LD. The rs81355859 variant was the most significantly associated SNP for the content of C18:2(n-6) (*p*-value = 3.01 × 10^−7^), and the PUFA (*p*-value = 4.96 × 10^−7^) and MUFA/PUFA (*p*-value = 9.55 × 10^−7^) ratios in the backfat of the BC1_LD animals. Nonetheless, despite the overlap between the significantly associated regions of BC1_PI (0–10.77 Mb) and BC1_LD (2.06–10.55 Mb) on SSC2 (Fig. 3 and Supplementary Table S3), no association was found on the BC1_PI-specific GWAS between the *FADS2*-SNPs and the three traits (C18:2(n-6), PUFA and MUFA/PUFA) that the two SSC2 QTLs of both backcrosses have in common. Altogether, it is unlikely that any of the five genotyped SNPs of the *FADS2* gene are the causal mutation for explaining the SSC2 QTL in our population. Thus, further research is needed to analyse other variants or even variants in other candidate genes that could be the causal mutation of this QTL.

#### ELOVL6

GWAS for the percentages of C14:0 (Fig. 1A), C16:0 (Fig. 1B), and C16:1(n-7) (Fig. 1C) in backfat were reanalysed including seven SNPs of the *ELOVL6* gene. The seven SNPs located in *ELOVL6* have previously been described in the BC1_LD animals^17,66^, four of them were upstream variants located in the proximal promoter region of *ELOVL6* (rs325491325, rs345025813 (*ELOVL6:c.-533C*>*T*), rs341847499 and rs322956047 (*ELOVL6:c.-394G*>*A*)), one was located on the fourth exon (*ELOVL6:c.416C*>*T*), and two were located in the fifth exon (3′-UTR) (*ELOVL6:c.1408C*>*T* and *ELOVL6:c.1922C*>*T*). In accordance with previous works in the BC1_LD pigs^66^, *ELOVL6:c.-394G*>*A* was the most associated polymorphism with the percentages of C16:0 (*p*-value = 1.19 × 10^−12^) and C16:1(n-7) (*p*-value = 2.02 × 10^−7^) in backfat (Supplementary Table S4). As stated before, previous studies in our group provided genetic and functional evidence that supported the *ELOVL6:c.-394G*>*A* polymorphism as the causal mutation for the QTL on SSC8 affecting the percentages of C16:0 and C16:1(n-7) in backfat and IMF of BC1_LD animals^17,66^.

In addition, in our study *ELOVL6:c.-394G*>*A* SNP was also the most associated polymorphism for the abundance of C14:0 (*p*-value = 4.55 × 10^−8^) in backfat. Apart from the *ELOVL6:c.-394G*>*A* polymorphism, other three *ELOVL6*-SNPs (*ELOVL6:c.-533C*>*T, ELOVL6:c.416C*>*T* and *ELOVL6:c.1922C*>*T*) were also significantly associated with the percentages of C14:0, C16:0, and C16:1(n-7) in backfat (Supplementary Table S4). The *ELOVL6:c.-394G*>*A* was not segregating in the BC1_DU and BC1_PI backcrosses (Supplementary Table S5) and no region was found associated on SSC8 for the BC1_DU and BC1_PI pigs in the backcross-specific GWAS (Supplementary Table S3). The *p*-values obtained for the *ELOVL6:c.-394G*>*A* polymorphism in the backcross-specific GWAS performed in the BC1_LD were closer to the *p*-values obtained in the merged dataset GWAS for the percentages of C14:0 (*p*-value = 1.42 × 10^−8^), C16:0 (*p*-value = 5.12 × 10^−15^), and C16:1(n-7) (*p*-value = 1.56 × 10^−8^) in backfat. Overall, our results support the *ELOVL6:c.-394G*>*A* polymorphism as the causal mutation of SSC8 QTL for C14:0, C16:0, and C16:1(n-7) percentages in backfat in BC1_LD animals. The A allele of the *ELOVL6:c.-394G*>*A* SNP in the BC1_LD animals was probably maternally inherited from the Landrace sows. In the BC1_LD, the G allele is the predominant allele with a frequency of 0.64 and it is associated with a lower expression of the *ELOVL6* gene in comparison to the A allele^66^. Hence, an impairment or reduction on the *ELOVL6* function produces the accumulation of C16:0 and C16:1(n-7) in muscle and adipose tissue^17,118^, which can modify meat quality.

#### FASN

GWAS for the percentage of C14:0 in backfat was reanalysed including one SNP from the upstream region of the *FASN* gene (rs327036596), which was found in the genome sequences of the BC1_LD founders^117^. However, rs327036596 was not associated with the percentage of C14:0 in backfat in the merged dataset neither in the backcross-specific GWAS. Furthermore, rs327036596 was not segregating in the BC1_DU animals (Supplementary Table S5), although a genomic region (0–7.86 Mb) of SSC12 was found in association for the same trait (C14:0 in backfat) in the BC1_DU backcross (Supplementary Table S3). Therefore, other variants of *FASN* could be the causal mutation of this QTL. Such potential candidate may be rs324640280, which has been found in association with the C14:0 content in backfat as well as with other FAs^119^.

#### ELOVL7

GWAS for the C20:1(n-9)/C20:0 ratio in backfat was reanalysed including three SNPs of the *ELOVL7* gene (Fig. 2) found in the genome sequences of the BC1_LD founders^117^. One of the three SNPs was an upstream variant (rs322657523) and the other two SNPs were located in the 5′-UTR (rs343494956 (*ELOVL7:c.-46A*>*G*)) and the 3′-UTR (tenth exon) (rs325490947 (ELOVL7:c.*1432A>*G*)). The SNP located in the tenth exon of *ELOVL7* (ELOVL7:c.*1432A>*G*) was the only significantly associated *ELOVL7-*SNP (*p*-value = 1.48 × 10^−7^) (Supplementary Table S4), but other two SNPs showed a lower *p*-value, rs81297480 and rs81458871 (both *p*-values = 2.66 × 10^−8^). Therefore, the ELOVL7:c.*1432A>*G* SNP is unlikely to be the causal mutation of the SSC16 QTL for the variation of the C20:1(n-9)/C20:0 ratio in backfat. In addition, ELOVL7:c.*1432A>*G* was not segregating in the BC1_DU pigs and no GG individuals were observed in the BC1_PI pigs (Supplementary Table S5). The higher significance (*p*-value = 1.48 × 10^−7^) obtained in the merged dataset GWAS for the ELOVL7:c.*1432A>*G* SNP than the significance (*p*-value = 3.16 × 10^−5^) obtained in the BC1_LD-specific GWAS was probably due to the inclusion of the heterozygous BC1_PI individuals in the analysis of the merged dataset. Nonetheless, these findings suggest that the *ELOVL7* gene is a clear candidate to explain the differences in the C20:1(n-9)/C20:0 ratio and further studies will be required to find the causal mutation.

#### SCD

GWAS for the C18:1(n-9)/C18:0 and MUFA/SFA ratios in IMF were reanalysed including five SNPs of SSC14 of the *SCD* gene. Of the five SNPs, rs323081995 (*SCD:c.-353T*>*C*) and rs80912566 (*SCD:c.-233C*>*T*) were described by Estany et al.^120^ and located in the 5′-UTR, while the other three variants (rs338494000, rs710198292 and rs331969256) located in the 3′-UTR of *SCD* were found in the BC1_LD founders^117^. On the GWAS conducted in the merged dataset, no significant signal was found for any of the five *SCD*-variants. Nonetheless, on the BC1_DU-specific GWAS, two *SCD*-SNPs (*SCD:c.-353T*>*C* and *SCD:c.-233C*>*T*) were found significantly associated with the C18:1(n-9)/C18:0 (*p*-values = 2.18 × 10^−6^ and 1.52 × 10^−5^, respectively) and MUFA/SFA (*p*-values = 1.80 × 10^−5^ and 5.54 × 10^−5^, respectively) ratios in IMF. Furthermore, in accordance with the literature^120^, *SCD:c.-233C*>*T* was proposed as the causal mutation for explaining part of the genetic variance for the desaturation ratios (18:1/18:0 and MUFA/SFA) in muscle. However, the most significant SNPs of the SSC14 region in BC1_DU were rs335655209 (*p*-value = 2.39 × 10^−7^) for the C18:1(n-9)/C18:0 ratio and rs80948585 (*p*-value = 8.71 × 10^−7^) for the MUFA/SFA ratio. No region was found associated on SSC14 for the BC1_LD and BC1_PI backcrosses (Supplementary Table S3). This result suggests that the causal mutation of SSC14 QTL for the C18:1(n-9)/C18:0 and MUFA/SFA ratios in IMF may be other uncharacterized genetic variants of *SCD*, which is in accordance with another GWAS study performed for backfat FA composition in a Duroc population^24^, and further studies are warranted to identify them.

## Conclusions

Our results increase the knowledge of the genetic basis of FA composition and lipid metabolism. We have described fifteen regions of the pig genome that are associated with fat composition traits in adipose tissue and muscle in three different backcrosses with the Iberian breed in common. Despite some regions and candidate genes have been reported in accordance with previous association studies, we have identified new regions and candidate genes that had not been described yet. In total, 52 candidate genes were proposed. This list of candidate genes might be useful for selection of specific FA composition traits in meat of commercial pig breeds. In addition, we genotyped and analysed 21 polymorphisms of candidate genes *FADS2*, *FASN*, *ELOVL6*, *ELOVL7* and *SCD*. These polymorphisms can be used as genetic markers for meat quality selection of commercial pig breeds, although some polymorphisms were breed-specific, and further analyses are warranted to find and evaluate possible causal mutations.

## Supplementary information

Supplementary Information 1.

Supplementary Information 2.

Supplementary Information 3.

Supplementary Information 4.

Supplementary Information 5.

Supplementary Information 6.

## Data Availability

The datasets used and analysed during the current study are available from the corresponding author on reasonable request.
