# Supplementary material for: Identification of strong candidate genes for backfat and intramuscular fatty acid composition in three crosses based on the Iberian pig
Source: Sci Rep. 2020 Aug 18;10:13962. doi: 10.1038/s41598-020-70894-2 (PMC7435270; doi:10.1038/s41598-020-70894-2)
Supplement: Supplementary file 6 — Supplementary Information 6. [file 41598_2020_70894_MOESM6_ESM.pdf]

Identification of strong candidate genes for backfat and intramuscular fatty acid composition in three crosses based on the Iberian pig

Daniel Crespo-Piazuelo[1,2,*], Lourdes Criado-Mesas[1], Manuel Revilla[1,2], Anna Castelló[1,2], José L. Noguera[3], Ana I. Fernández[4], Maria Ballester[5] and Josep M. Folch[1,2]

[1] Plant and Animal Genomics, Centre for Research in Agricultural Genomics (CRAG), CSIC-IRTA-UAB-UB Consortium, 08193 Bellaterra, Spain

[2] Departament de Ciència Animal i dels Aliments, Facultat de Veterinària, Universitat Autònoma de Barcelona (UAB), 08193 Bellaterra, Spain

[3] Genètica i Millora Animal, Institut de Recerca i Tecnologia Agroalimentàries (IRTA), 25198 Lleida, Spain

[4] Departamento de Mejora Genética Animal, Instituto Nacional de Investigación y Tecnología Agraria y Alimentaria (INIA), 28040 Madrid, Spain

[5] Departament de Genètica i Millora Animal, Institut de Recerca i Tecnologia Agroalimentàries (IRTA), 08140 Caldes de Montbui, Spain

*E-mail: daniel.crespo@cragenomica.es

Supplementary Information S1. Quantile-quantile (Q-Q) plot of the association analysis between the 18 traits in backfat (BF) or *longissimus dorsi* muscle (LD) with significant SNPs. Trait and tissue are specified on the title of each figure. Red and blue lines indicate those SNPs that are below the genome-wide significance threshold (FDR≤0.05 and FDR≤0.1, respectively). Genomic inflation factor (λ) is indicated in the legend.

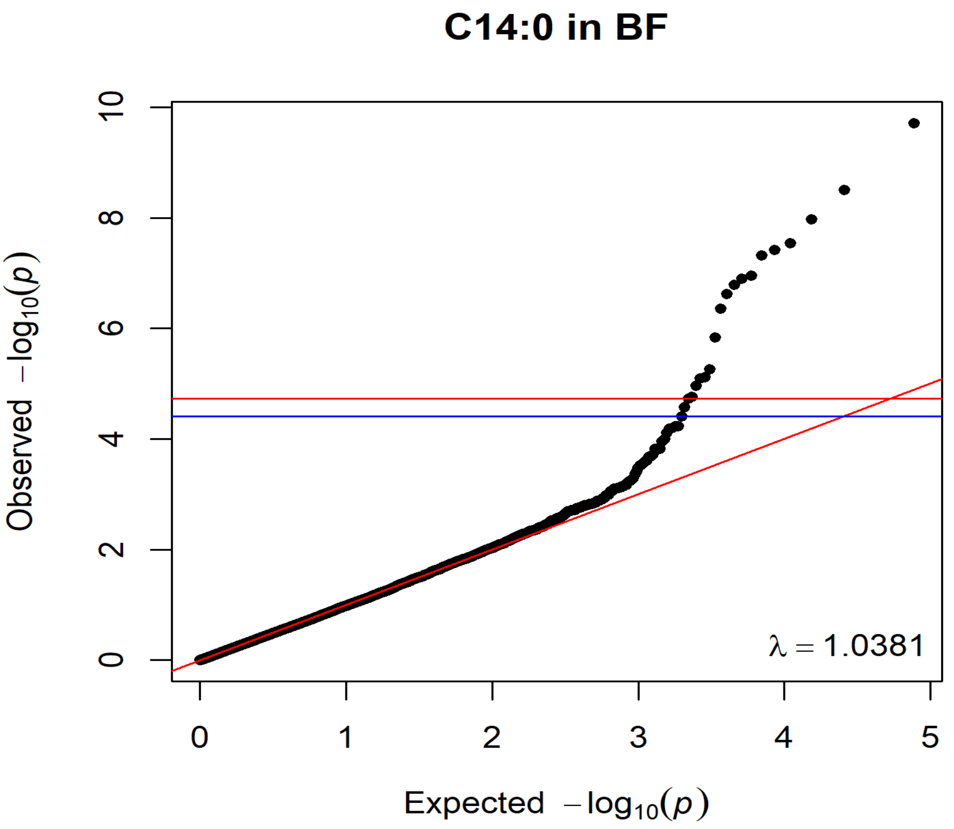
C14:0 in BF
Observed −log10(p)
Expected −log10(p)
λ = 1.0381
0
2
4
6
8
10
0
1
2
3
4
5

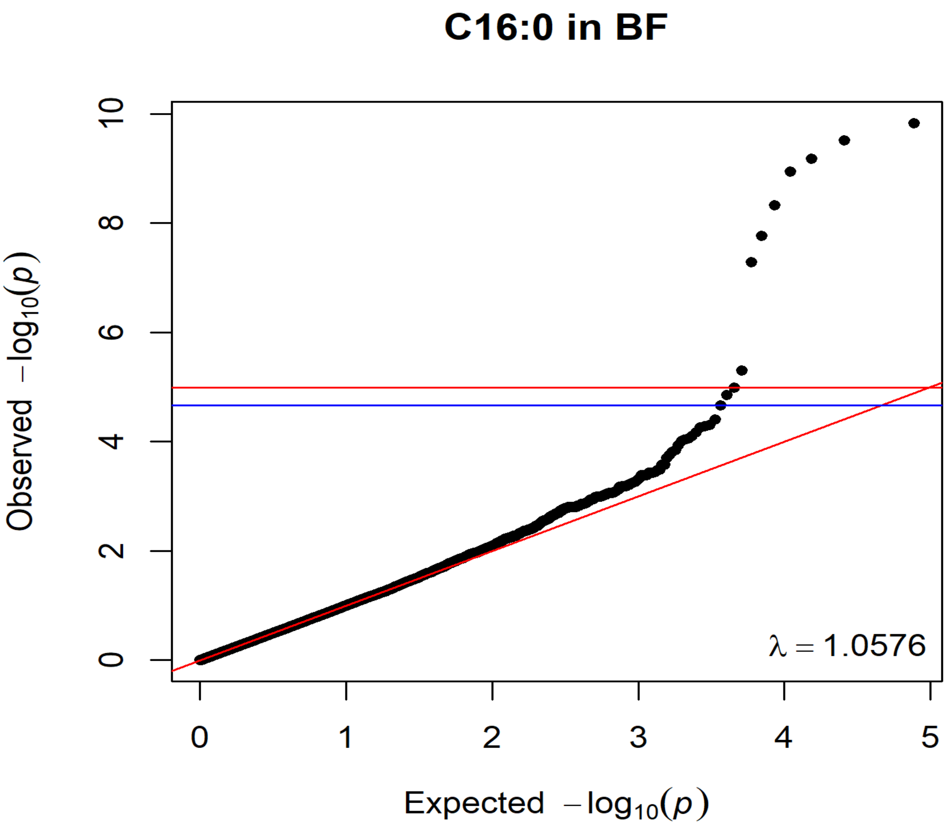

C16:0 in BF
Observed −log10(p)
Expected −log10(p)
λ = 1.0576
0
2
4
6
8
10
0
1
2
3
4
5

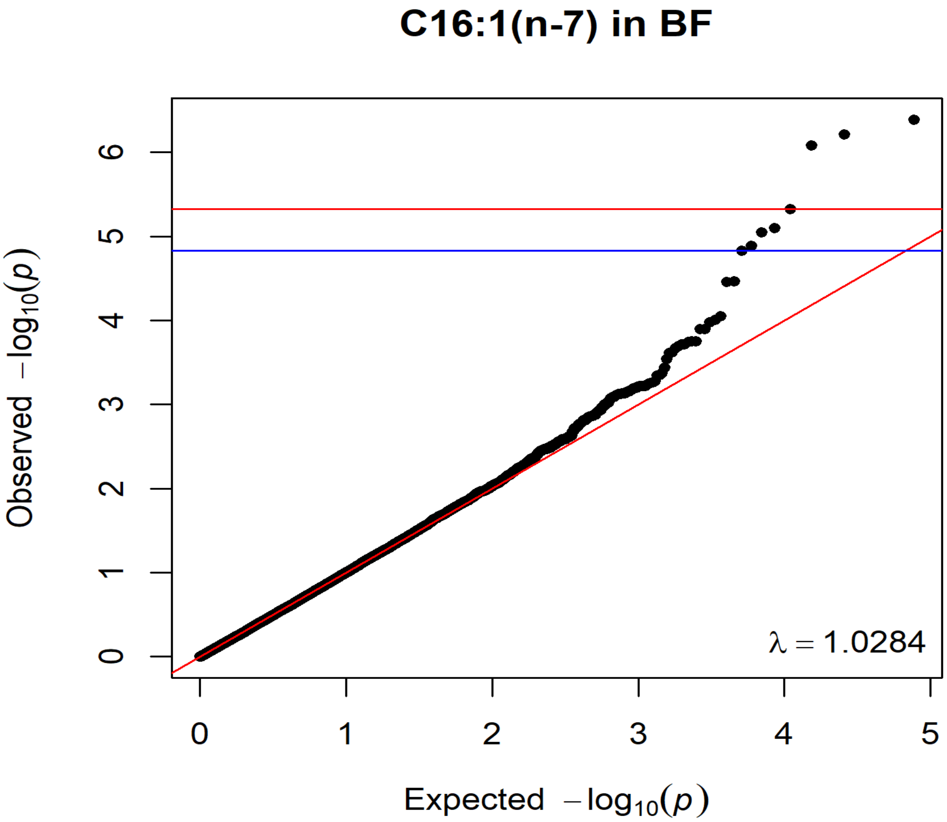

C16:1(n-7) in BF
Observed $-\log_{10}(p)$
Expected $-\log_{10}(p)$
0
1
2
3
4
5
6
0
1
2
3
4
5
$\lambda = 1.0284$

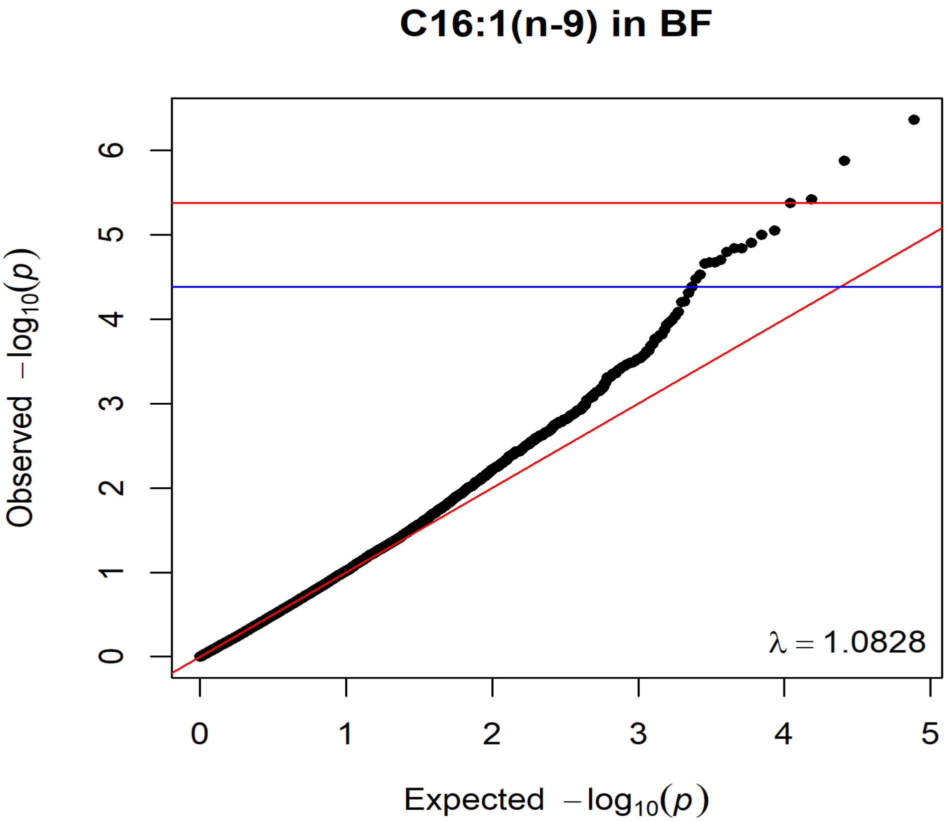

C16:1(n-9) in BF
Observed $-\log_{10}(p)$
Expected $-\log_{10}(p)$
$\lambda = 1.0828$
0
1
2
3
4
5
6

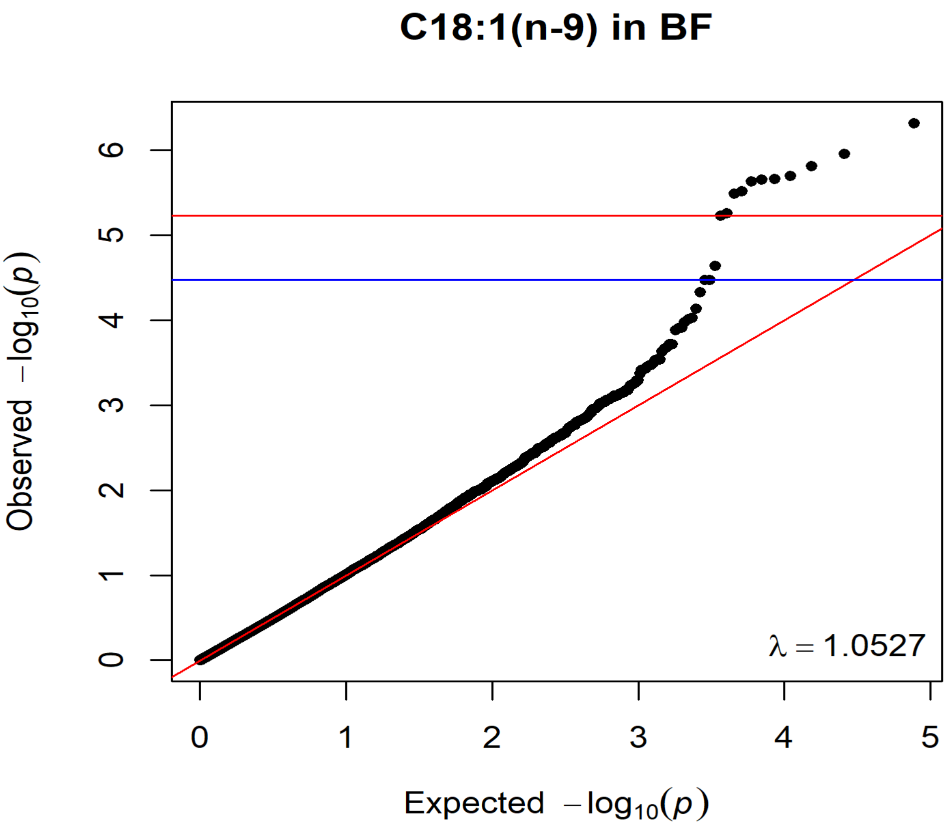
C18:1(n-9) in BF
Observed −log10(p)
Expected −log10(p)
λ = 1.0527
0
1
2
3
4
5
6
0
1
2
3
4
5

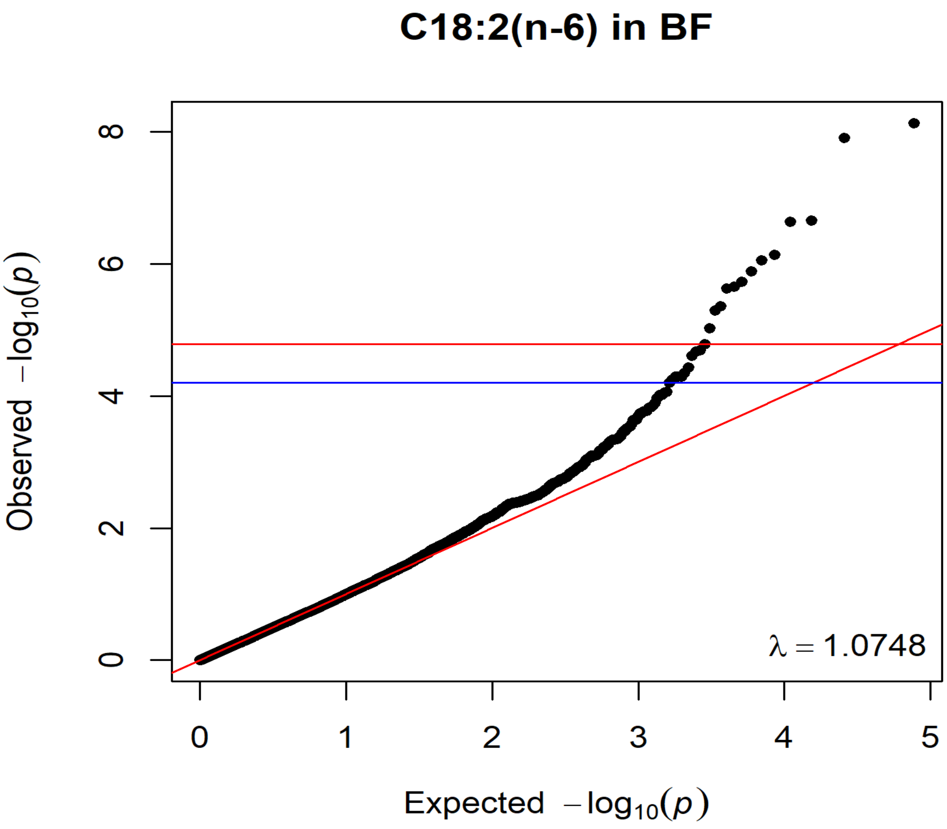

C18:2(n-6) in BF
Observed −log10(p)
Expected −log10(p)
λ = 1.0748
0
1
2
3
4
5
0
2
4
6
8

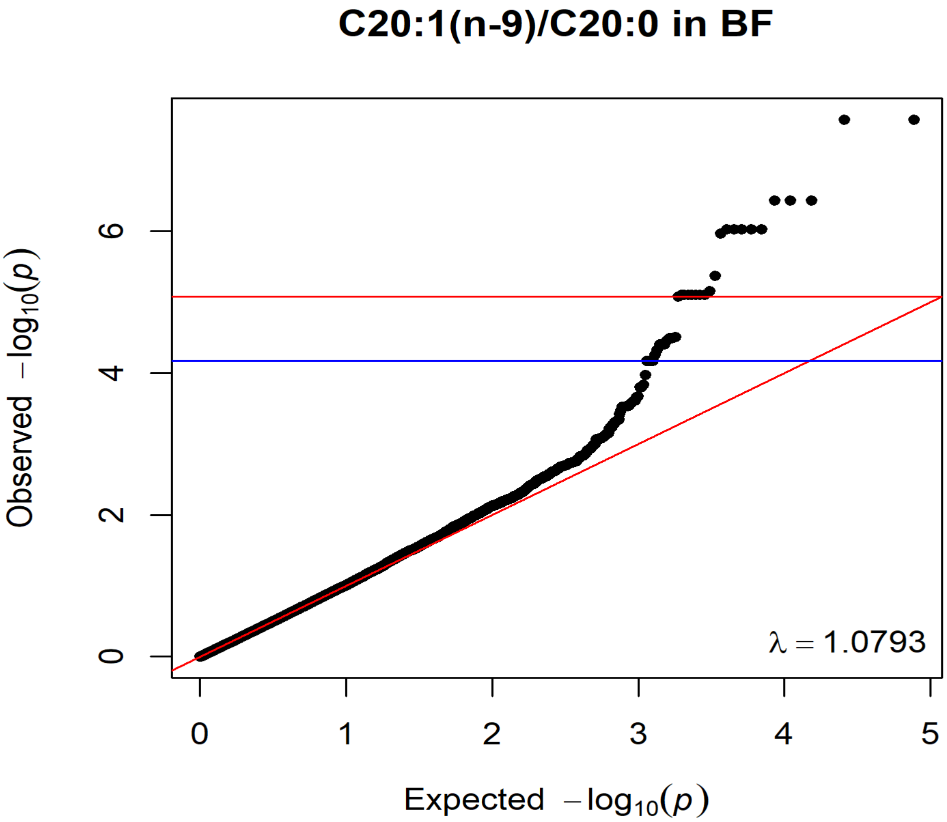

C20:1(n-9)/C20:0 in BF
Observed $-\log_{10}(p)$
Expected $-\log_{10}(p)$
$\lambda = 1.0793$
0
2
4
6
0
1
2
3
4
5

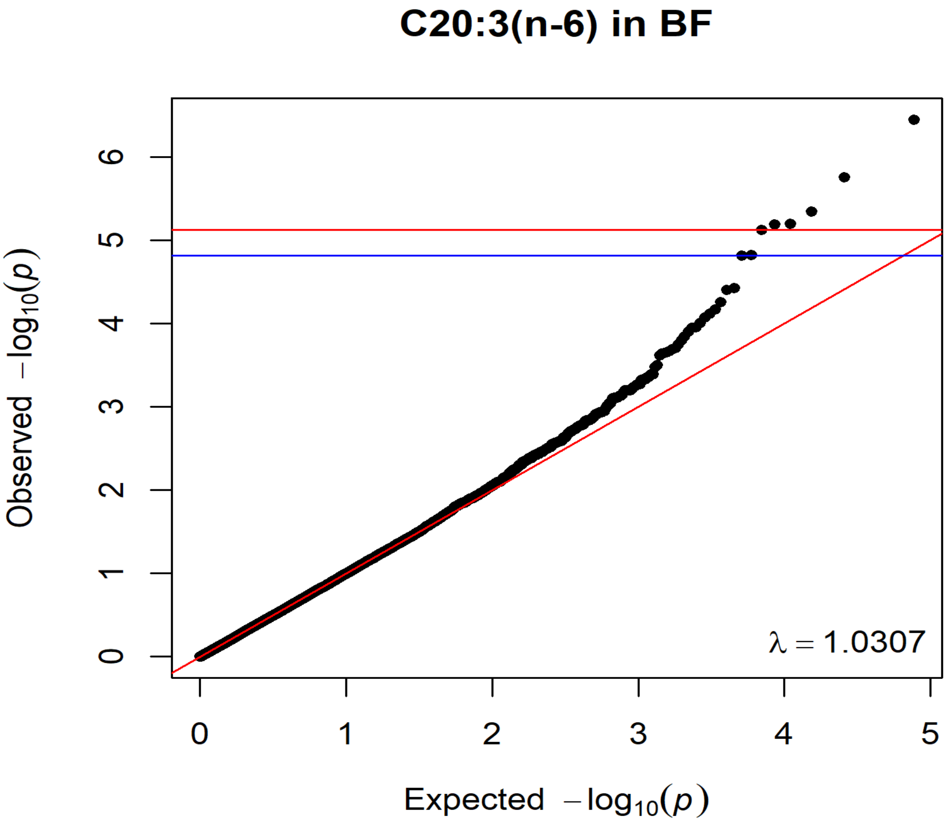
C20:3(n-6) in BF
Observed $-\log_{10}(p)$
Expected $-\log_{10}(p)$
$\lambda = 1.0307$
0
1
2
3
4
5
6

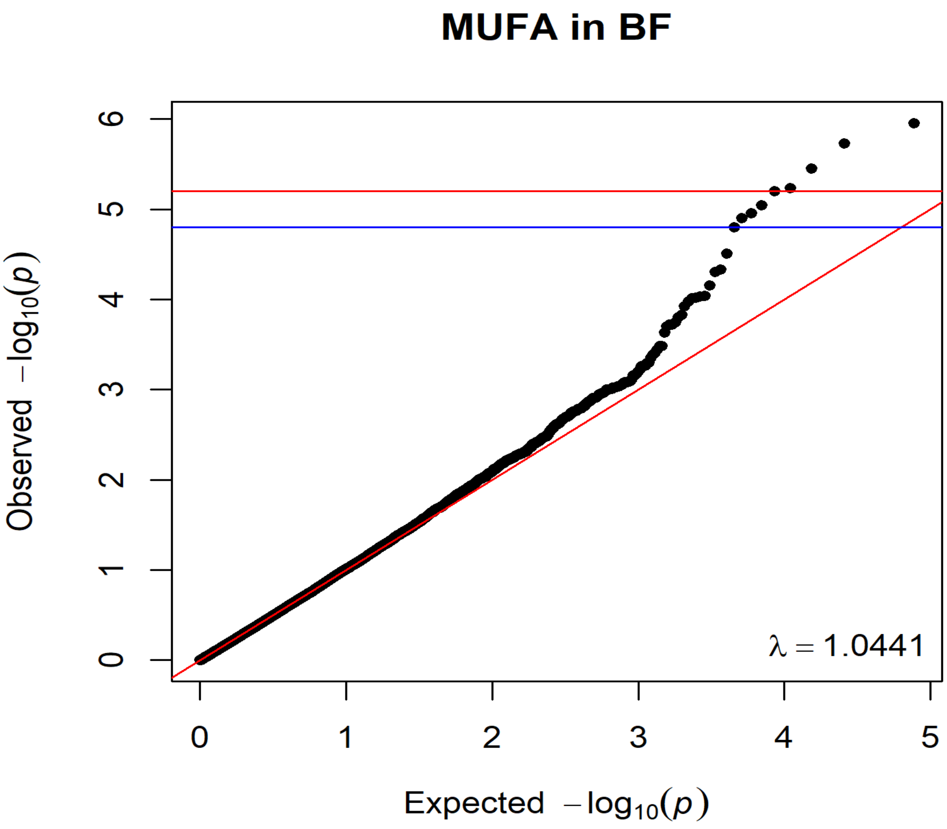
MUFA in BF
Observed $-\log_{10}(p)$
Expected $-\log_{10}(p)$
0
1
2
3
4
5
6
0
1
2
3
4
5
$\lambda = 1.0441$

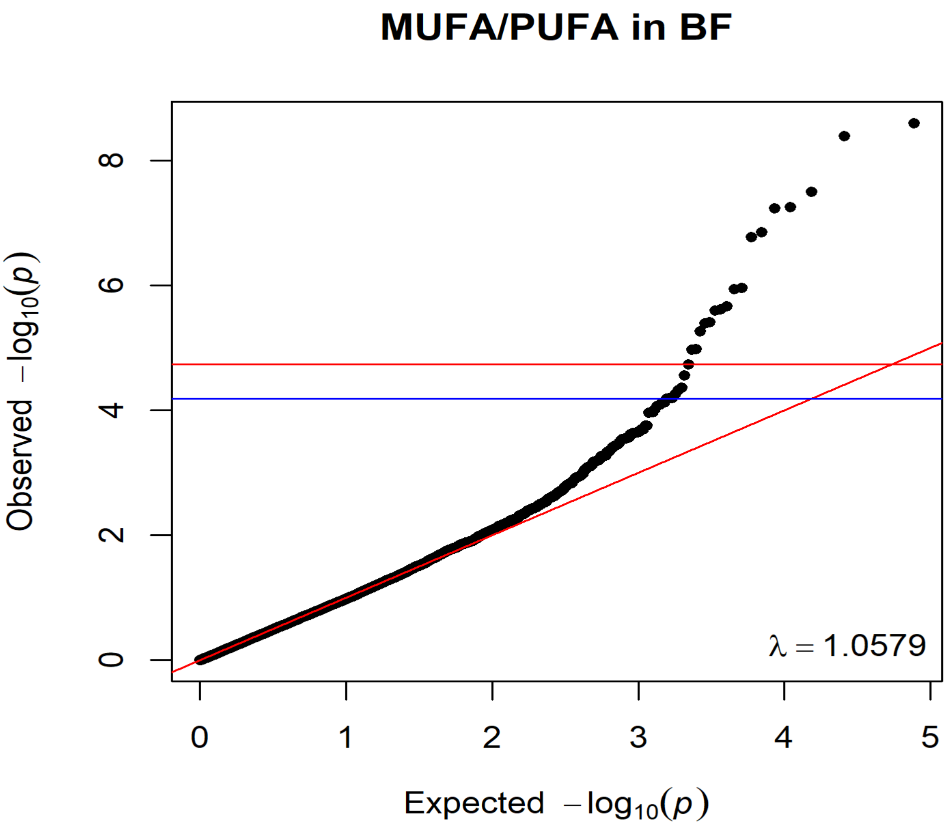
MUFA/PUFA in BF
Observed −log10(p)
Expected −log10(p)
λ = 1.0579
0
2
4
6
8
0
1
2
3
4
5

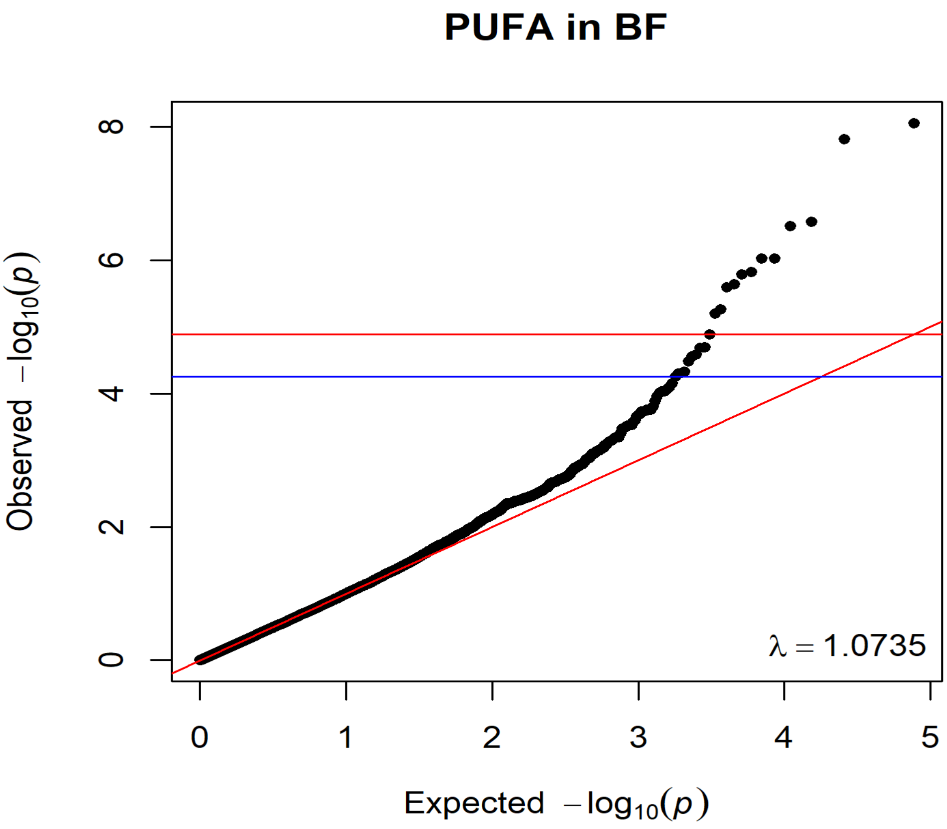
PUFA in BF
Observed −log10(p)
Expected −log10(p)
λ = 1.0735
0
2
4
6
8
0
1
2
3
4
5

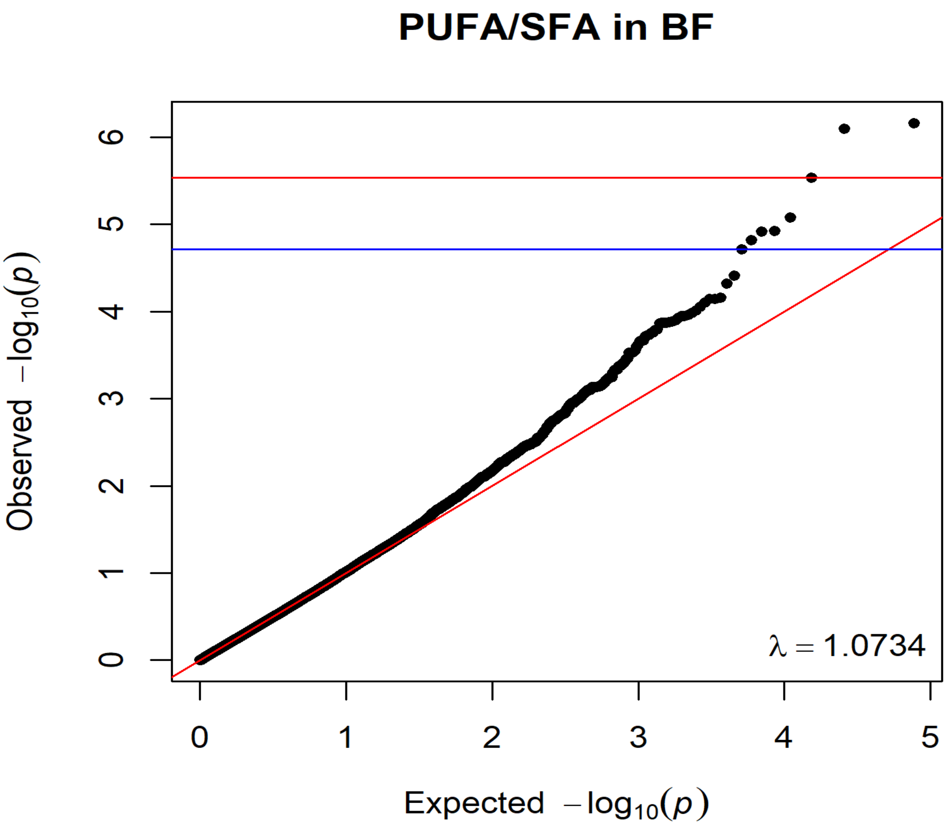
PUFA/SFA in BF
Observed $-\log_{10}(p)$
Expected $-\log_{10}(p)$
$\lambda = 1.0734$
0
1
2
3
4
5
6

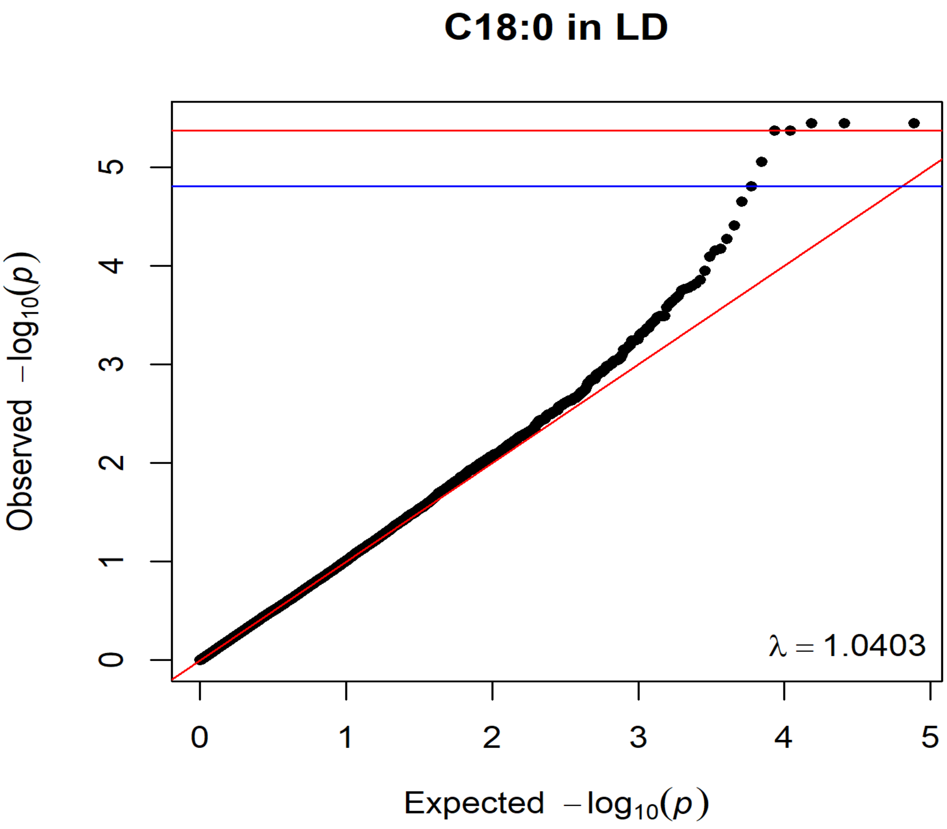
C18:0 in LD
Observed $-\log_{10}(p)$
Expected $-\log_{10}(p)$
0
1
2
3
4
5
0
1
2
3
4
5
$\lambda = 1.0403$

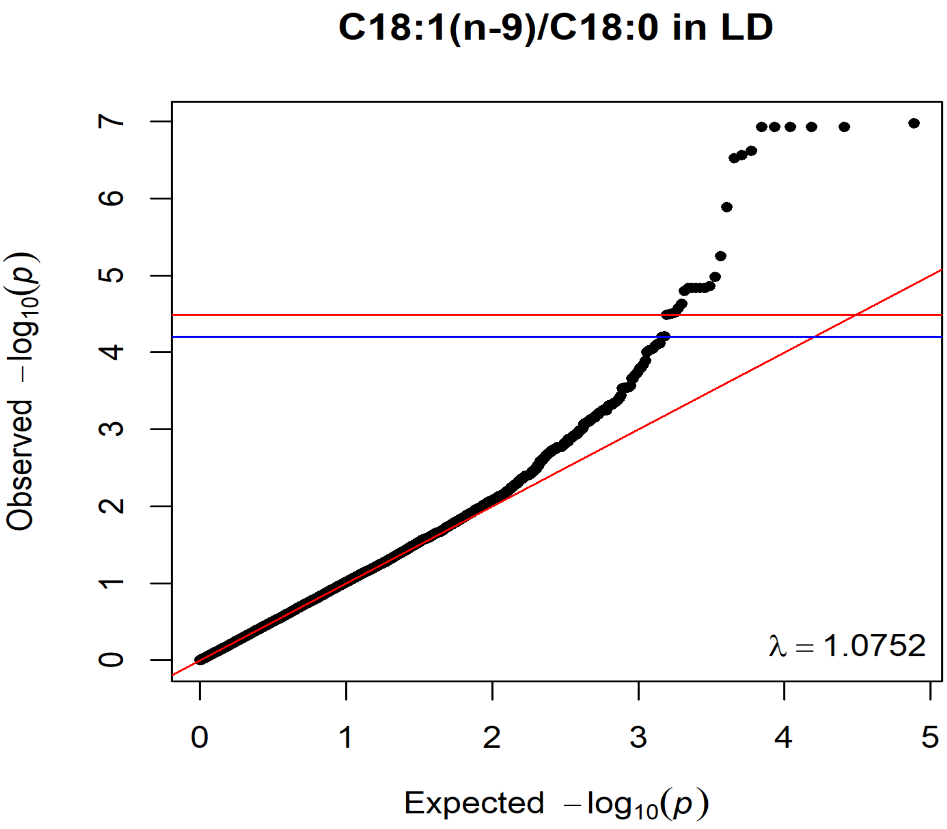
C18:1(n-9)/C18:0 in LD
Observed $-\log_{10}(p)$
Expected $-\log_{10}(p)$
$\lambda = 1.0752$
0
1
2
3
4
5
6
7

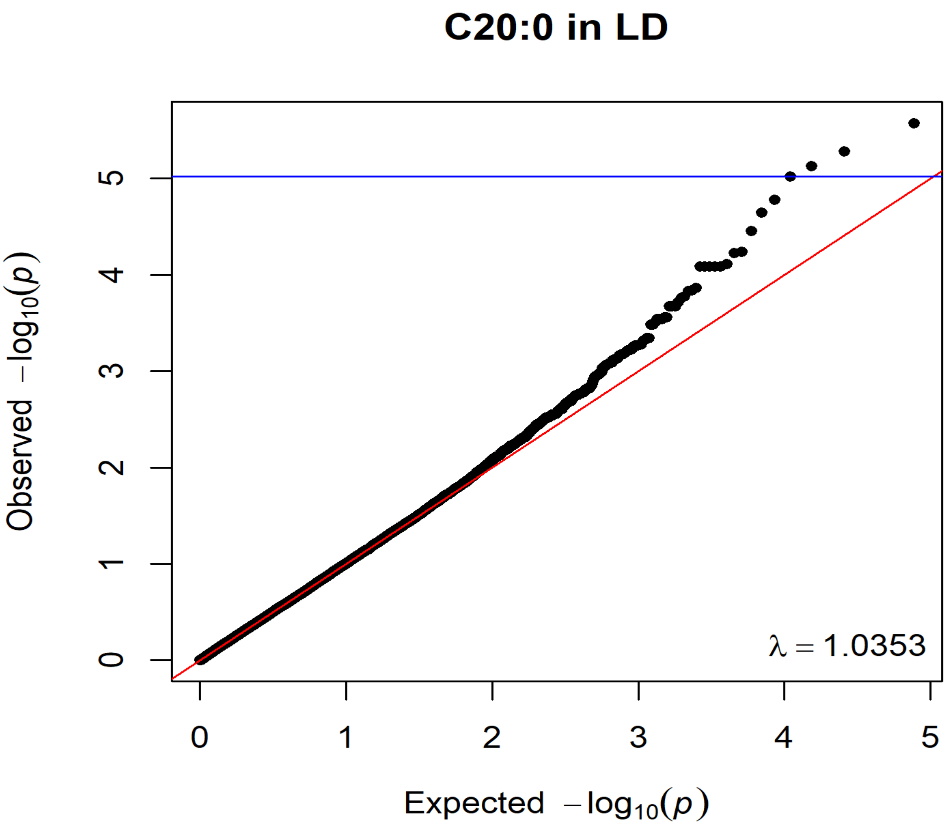

C20:0 in LD
Observed −log10(p)
Expected −log10(p)
λ = 1.0353
0
1
2
3
4
5

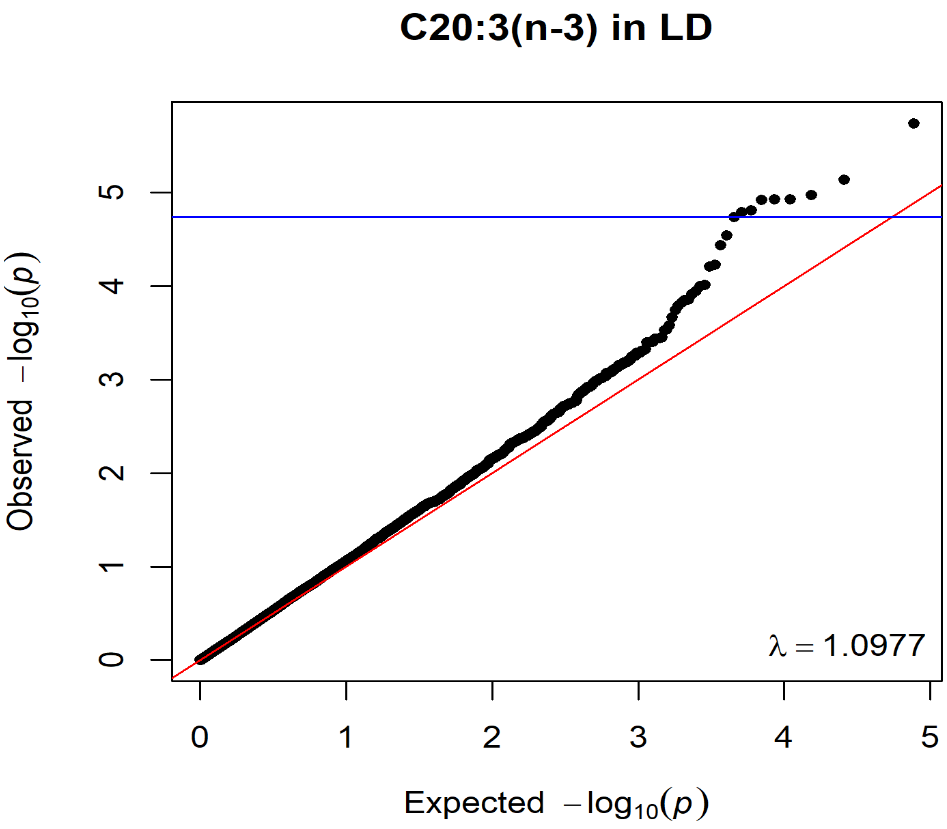
C20:3(n-3) in LD
Observed −log10(p)
Expected −log10(p)
λ = 1.0977
0
1
2
3
4
5

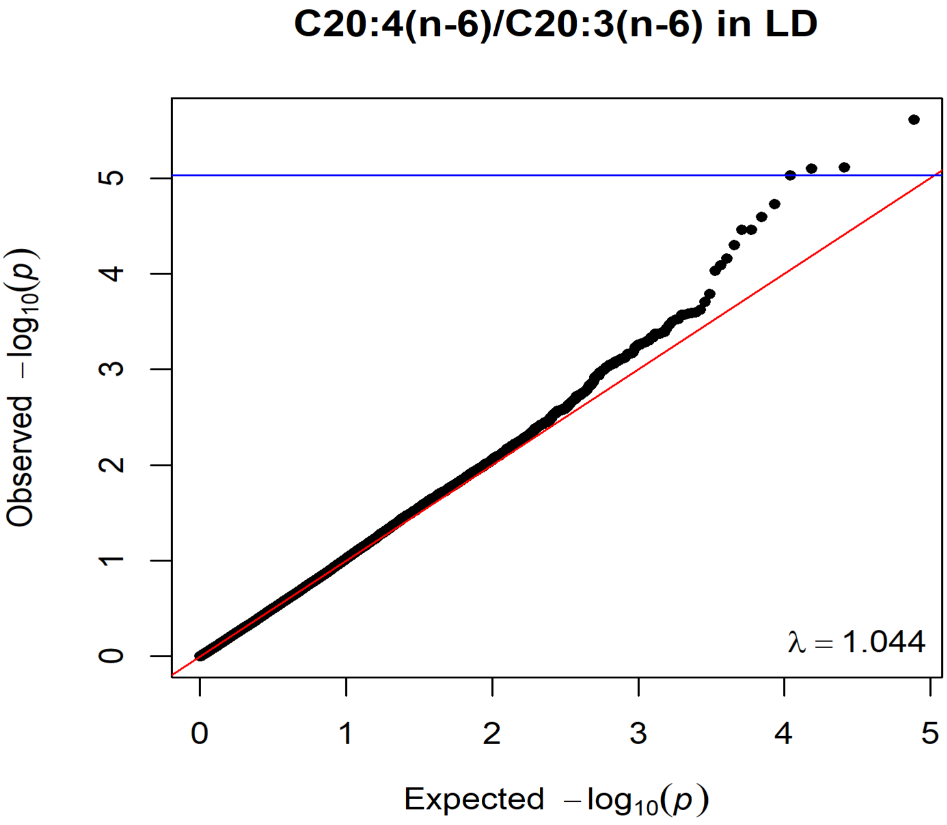
C20:4(n-6)/C20:3(n-6) in LD
Observed −log10(p)
Expected −log10(p)
λ = 1.044
0
1
2
3
4
5

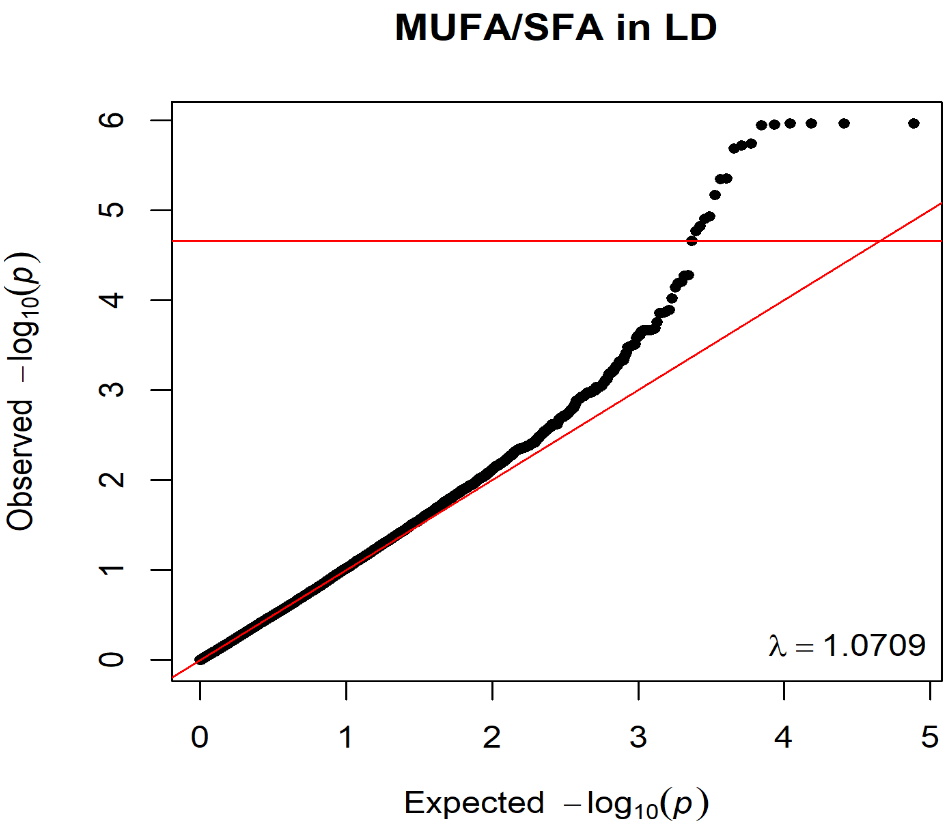
MUFA/SFA in LD
Observed $-\log_{10}(p)$
Expected $-\log_{10}(p)$
0
1
2
3
4
5
6
0
1
2
3
4
5
$\lambda = 1.0709$
